# The Effect of Training Sample Size on the Prediction of White Matter Hyperintensity Volume in a Healthy Population Using BIANCA

**DOI:** 10.3389/fnagi.2021.720636

**Published:** 2022-01-11

**Authors:** Niklas Wulms, Lea Redmann, Christine Herpertz, Nadine Bonberg, Klaus Berger, Benedikt Sundermann, Heike Minnerup

**Affiliations:** ^1^Institute of Epidemiology and Social Medicine, University of Muenster, Muenster, Germany; ^2^Clinic of Radiology, University Hospital Muenster, Muenster, Germany; ^3^Institute of Radiology and Neuroradiology, Evangelisches Krankenhaus, Medical Campus, University of Oldenburg, Oldenburg, Germany; ^4^Research Center Neurosensory Science, University of Oldenburg, Oldenburg, Germany

**Keywords:** white matter hyperintensities, fully-automated segmentation, neuroimaging marker, lesion segmentation, population studies

## Abstract

**Introduction:** White matter hyperintensities of presumed vascular origin (WMH) are an important magnetic resonance imaging marker of cerebral small vessel disease and are associated with cognitive decline, stroke, and mortality. Their relevance in healthy individuals, however, is less clear. This is partly due to the methodological challenge of accurately measuring rare and small WMH with automated segmentation programs. In this study, we tested whether WMH volumetry with FMRIB software library v6.0 (FSL; https://fsl.fmrib.ox.ac.uk/fsl/fslwiki) Brain Intensity AbNormality Classification Algorithm (BIANCA), a customizable and trainable algorithm that quantifies WMH volume based on individual data training sets, can be optimized for a normal aging population.

**Methods:** We evaluated the effect of varying training sample sizes on the accuracy and the robustness of the predicted white matter hyperintensity volume in a population (*n* = 201) with a low prevalence of confluent WMH and a substantial proportion of participants without WMH. BIANCA was trained with seven different sample sizes between 10 and 40 with increments of 5. For each sample size, 100 random samples of T1w and FLAIR images were drawn and trained with manually delineated masks. For validation, we defined an internal and external validation set and compared the mean absolute error, resulting from the difference between manually delineated and predicted WMH volumes for each set. For spatial overlap, we calculated the Dice similarity index (SI) for the external validation cohort.

**Results:** The study population had a median WMH volume of 0.34 ml (IQR of 1.6 ml) and included *n* = 28 (18%) participants without any WMH. The mean absolute error of the difference between BIANCA prediction and manually delineated masks was minimized and became more robust with an increasing number of training participants. The lowest mean absolute error of 0.05 ml (SD of 0.24 ml) was identified in the external validation set with a training sample size of 35. Compared to the volumetric overlap, the spatial overlap was poor with an average Dice similarity index of 0.14 (SD 0.16) in the external cohort, driven by subjects with very low lesion volumes.

**Discussion:** We found that the performance of BIANCA, particularly the robustness of predictions, could be optimized for use in populations with a low WMH load by enlargement of the training sample size. Further work is needed to evaluate and potentially improve the prediction accuracy for low lesion volumes. These findings are important for current and future population-based studies with the majority of participants being normal aging people.

## 1. Introduction

White matter hyperintensities of presumed vascular origin (WMH) are a common finding in MRI using fluid attenuation inversion recovery (FLAIR) sequences in older subjects (Wardlaw et al., 2015). In patients with cardiovascular disease, severe confluent WMH is an important imaging marker of cerebral small vessel disease associated with an increased risk of stroke, dementia, and mortality (Debette and Markus, 2010; Wardlaw et al., 2013). Also, in a minority of individuals, a distinct morphology or spatial distribution of WMH can hint toward an inflammatory disease of the central nervous system (Filippi et al., 2019). However, the clinical importance of mild to moderate WMH in otherwise healthy or younger subjects is less clear (Hopkins et al., 2006; Williamson et al., 2018).

Two main challenges impede the evaluation of the latter. First, clinical endpoints, such as a decline in cognitive function are subtle in a normal aging population and need to be repeatedly measured in a comprehensive fashion over a long period of time. Second, to assess the presence and analyze the progress of low WMH volumes, highly robust measurement methods are needed. In the beginnings, visual rating scales have been developed and frequently used in the past (Fazekas et al., 1987, 1993; Scheltens et al., 1993; Hopkins et al., 2006). These rating scales face many problems such as relatively low reliability in cohorts with low lesion loads (Wardlaw et al., 2004; Olsson et al., 2013), as well as ground and ceiling effects (Prins et al., 2004). Over the last years, several fully-automated tools have been developed, e.g., Brain Intensity AbNormality Classification Algorithm (BIANCA) (Griffanti et al., 2016), LST (Schmidt et al., 2012), OASIS (Sweeney et al., 2013), DeepMedic (Kamnitsas et al., 2016), nicMSlesions (Valverde et al., 2019), and the Rotterdam Scan Study Tool (de Boer et al., 2009). These tools have the advantage of scalability and standardization. They show high reliability when re-assessing the same subjects, but as many different tools and complex preprocessing pipelines are used, the reproducibility is still limited (Frey et al., 2019). Moreover, such tools are usually developed using data from populations with a high white matter hyperintensity load (Weeda et al., 2019) and are rarely tested in populations with low prevalence and the low average volume of WMH (Williamson et al., 2018).

The aim of this study was the evaluation of the automated WMH segmentation tool BIANCA (Griffanti et al., 2016) for the quantification of WMH volumes in a population with sporadic WMH and small average volumes. We particularly aimed to improve the training of the BIANCA lesion classifier under these circumstances. BIANCA (Griffanti et al., 2016) was chosen in this study, because of its release in the widely distributed FSL framework (Smith et al., 2004; Jenkinson et al., 2012) and the transparent and precise recommendations on data processing (Griffanti et al., 2016). A systematic review of several fully-automated tools further showed a reliable performance of BIANCA in an elderly cohort (Vanderbecq et al., 2020). One specific feature of BIANCA is the study- or scanner-specific training procedure of the algorithm on its own data. A minimum of 10 to 20 manually delineated white matter hyperintensity masks is recommended by the authors. However, the algorithm was originally trained on cardiovascular and neurodegenerative cohorts with a relatively high lesion load (Griffanti et al., 2016). Other authors recommend a k-value of 40 and used a sample size of 20 for training of a robust k-nearest neighbors (k-nn) delineation (Anbeek et al., 2004; Steenwijk et al., 2013).

We, therefore, evaluated the impact of increasing sample sizes for BIANCA on the accuracy and robustness of WMH prediction in a cohort with a low white matter hyperintensity load. We defined the accuracy of WMH prediction as minimizing the absolute error, i.e., the difference between the WMH volume of manually delineated masks and BIANCA predicted WMH volume on the single-observation level. Robustness was defined as the model-wise minimal mean absolute error per sample size. These measures were compared between seven training sample sizes for BIANCA, each resampled with 100 random draws without replacement from the study dataset. We used a sample of 201 images from the community-dwelling cohort of the BiDirect Study as a model for various ongoing population studies, e.g., the German National Cohort (Bamberg et al., 2015; Ahrens et al., 2020) or the UK Biobank (Alfaro-Almagro et al., 2018). We additionally calculated the Dice similarity index (SI) to evaluate the spatial overlap of manual and predicted WMH volumes for the external cohort.

## 2. Materials and Methods

### 2.1. Study Cohort

All data were collected as part of the BiDirect Study (Teismann et al., 2014; Teuber et al., 2017). This longitudinal study investigates the bidirectional association of subclinical cardiovascular disease and depression based on more than 2,000 participants who were repeatedly examined between 2010 and 2020 in Muenster, Germany. A population cohort [*n* = 912, 687 with MRI, at baseline (BL)], a depression cohort (n = 999, 732 with MRI, at BL), and a cardiovascular disease cohort (*n* = 347, 51 with MRI, at BL) were examined. The neuroimaging data used in this study originates from a subsample of the population cohort without clinical or imaging evidence of major neurological disease [*n* = 121 of 488 at second follow-up (FU)]. Manual white matter hyperintensity masks were first delineated in the MRI images of the second FU examination. Another *n* = 80 masks were then delineated in a random sample of these 121 participants in the corresponding BL images (BL, on average 6 years earlier), resulting in a total of *n* = 201 lesion masks.

### 2.2. Ethics

The study was approved by the Ethics Committee of the University of Muenster and the Westphalian Chamber of Physicians in Muenster, Germany. All participants gave their written informed consent.

### 2.3. MRI Data Acquisition

The following sequences were used from the MRI protocol of the BiDirect Study (Teuber et al., 2017): (1) 3D T1-weighted gradient echo sequence with inversion prepulse (TFE), TR: 7.26 ms, TE: 3.56 ms, TI: 404 ms, FA: 9°, matrix: 256 x 256, in-plane resolution (reconstructed): 1 x 1 mm, slices: 160, thickness: 2 mm (reconstructed to 1 mm slice thickness by zero filling in k-space), orientation: sagittal. (2) 2D fluid-attenuated inversion recovery sequence (FLAIR), TR: 11,000 ms, TE: 120, TI: 2,600, FA: 90°, matrix: 352 x 206 mm, FOV: 230 x 186, in-plane resolution (reconstructed): 0.45 x 0.45, slices: 27, thickness: 4 mm, inter-slice gap = 1 mm, orientation: axial; dimensions 512 x 512 x 27. All MR images were acquired using the same 3 Tesla MRI scanner (Intera with Achieva upgrade, Philips, Best, NL) using a transmit-receive head coil.

### 2.4. Manual Segmentation

**Figure 1:**
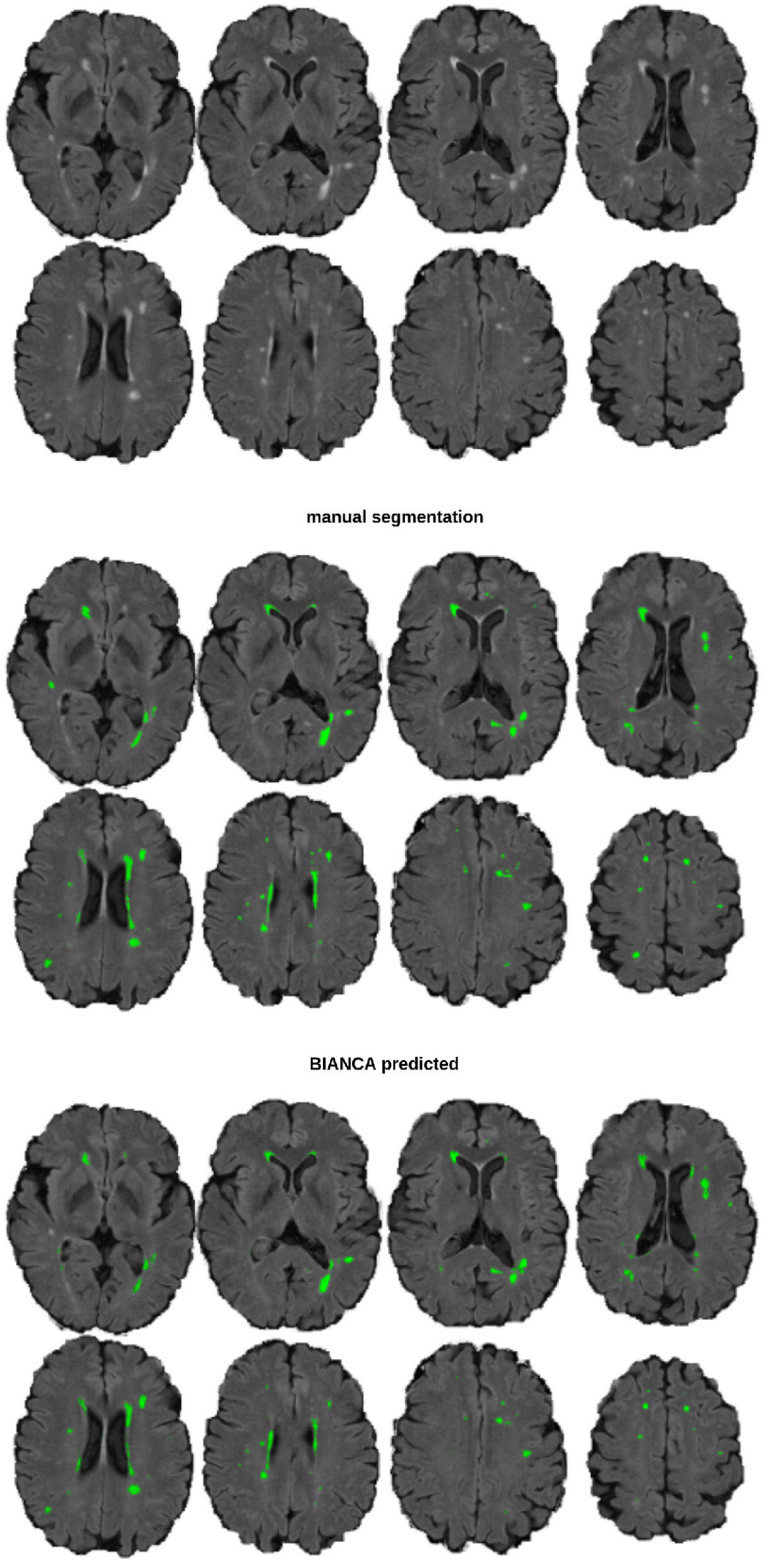
An example image of a participant with 5 ml WMH volume The top section shows the underlying FLAIR image, the middle section the manual segmentation mask, and the bottom section the BIANCA predicted mask (threshold: 0.8), from a model trained with a sample size of *n* = 40.

Manual white matter hyperintensity masks were segmented with FSLeyes (v0.22.1 McCarthy, 2019) using unprocessed FLAIR images (Figure 1). Two raters (CH, LR) were instructed and trained in manual delineation by an experienced radiologist (BS) and neurologist (HM). HM additionally viewed the segmented images for quality control to ensure the validity of the training procedure. The images were segmented interchangeably by one of the two raters, while the other one was present and took care, that the performance was according to the standard; in case of disagreements, those were *ad hoc* discussed between raters and if necessary, images were rated by case-based expert consensus meetings. In total, 201 images were segmented (80 from BL and 121 from FU).

### 2.5. Preprocessing Pipeline

The T1w images were reoriented and cropped using FSL (v6.0.3 Smith et al., 2004; Jenkinson et al., 2012). Then all T1w and FLAIR images were preprocessed using the fsl_anat pipeline. The bias corrected brain extracted images were used to register the T1w image to the FLAIR space using FLIRT (affine, 6 degrees of freedom). The bias corrected (non-brain extracted) FLAIR image was then masked with the T1w brain extraction mask (transformed to FLAIR space) to correct for minor misclassifications of the brain extraction on FLAIR images. From here on the brain extracted and bias corrected T1w and FLAIR images were used for BIANCA.

### 2.6. Sampling Strategy

**Figure 2:**
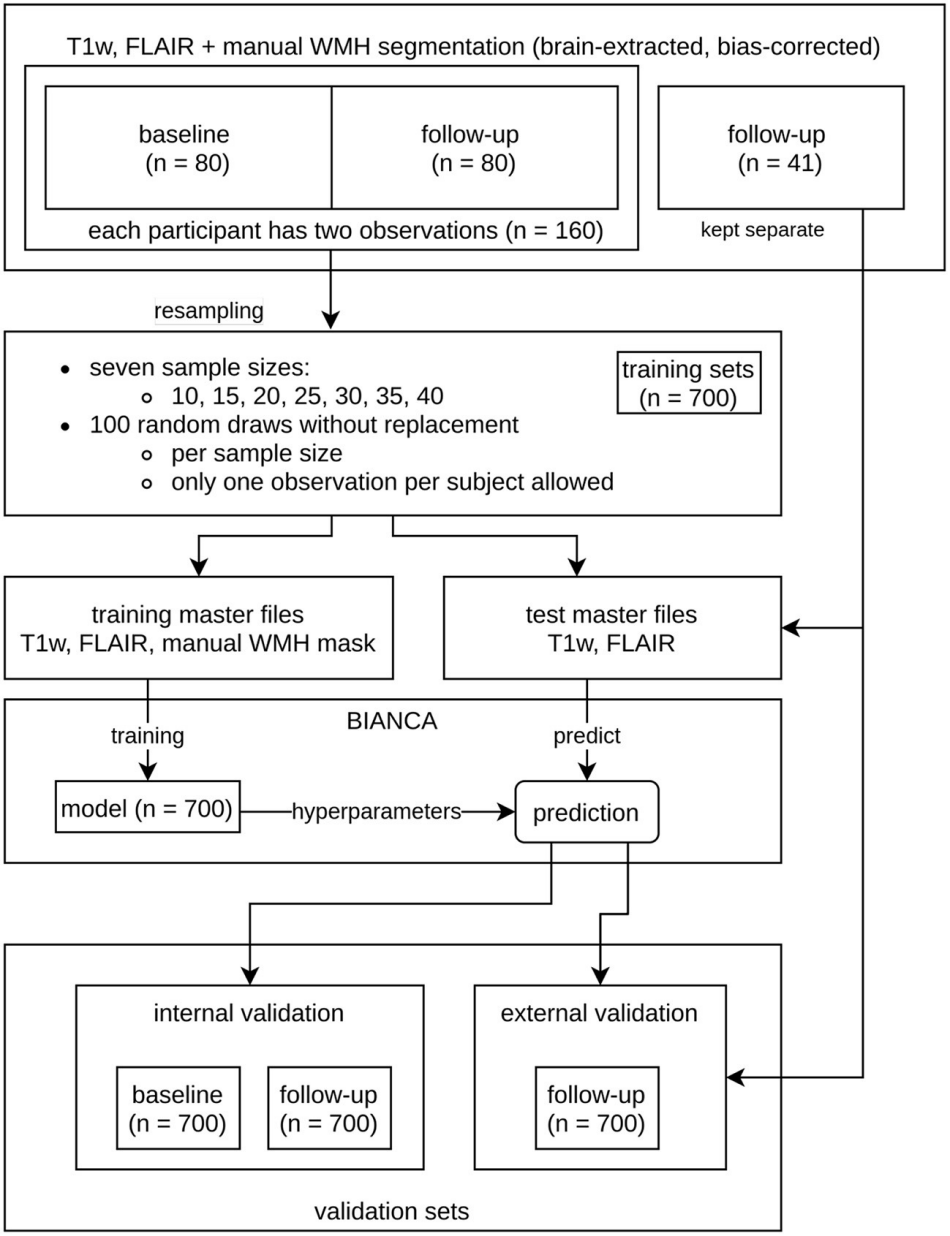
Overview of the resampling procedure for training the Brain Intensity AbNormality Classification Algorithm (BIANCA) models with varying sample sizes and evaluation of white matter hyperintensities (WMH) segmentation performance. The initial dataset consisted of the same 80 participants at baseline (BL) and follow-up (FU), as well as a separate external validation set with 41 participants. To prevent data leakage, all observations of a participant used in the training procedure were not allowed into the corresponding validation set. The resampling parameters are shown in the middle (7 sample sizes ranging from 10 to 40 with increments of 5; per sample size 100 random draws without replacement, only one observation per participant allowed). The corresponding validation sets (internal and external) are shown at the bottom.

To evaluate the impact of increasing training sample sizes on the accuracy and robustness of WMH prediction, training sets with seven different sample sizes (*n* = 10, 15, 20, 25, 30, 35, 40) were built (Figure 2, Tables 1, 2). For each training sample size, 100 random samples of T1w and FLAIR images were drawn without replacement from a set of 160 images (80 participants with BL and FU images) from the study dataset resulting in 700 different training sets.

**Table 1:** Description of datasets.

**Nomenclature**	**Resampled**	**Description**	**Used in training**
Study dataset	Static (*n* = 201)	Contains all data used for this publication	Only the resampling dataset
Resampling dataset	Static (*n* = 160)	Contains the subset of the study dataset, from which the participants are resampled for the training sets	Only the resampled ones
Training set	Random (*n* = 10 – *n* = 40)	Drawn from the resampling dataset, *n* = 100 per sample size	Everytime
Internal validation set	Depending on training set (*n* = 160 – 2 × sample size)	All images of participants, that are not in the corresponding training set	Never in the same draw to prevent data leakage
External validation set	Static (*n* = 41)	Contains the subset (*n* = 41) of the study dataset, that was never used for training	Never

**Table 2:** Number of observations in training and validation sets.

	**Validation sets**				
**Sampling**	**Internal validation**		**External validation**	**Predicted masks**	
**Training set (n)**	**BL (n)**	**FU (n)**	**FU (n)**	**Per model (n)**	**Overall (n)**
10	70	70	41	181	18,100
15	65	65	41	171	17,100
20	60	60	41	161	16,100
25	55	55	41	151	15,100
30	50	50	41	141	14,100
35	45	45	41	131	13,100
40	40	40	41	121	12,100

*Seven effective training sample sizes were used for resampling. For each sample size 100 random draws without replacement were conducted. To prevent data leakage, observations of the same subject were only used once per training and internal validation set, respectively. The resulting 700 prediction models (100 draws per training sample size (n = 7)) were applied to two types of validation data: The internal validation set comprises all participants that were not used for the corresponding training set. These comprise observations at BL and FU of each participant. The external validation set comprises 41 participants from FU that were never used for any training. In total (sum of the last column), n = 105,700 masks were predicted per threshold*.

As required by BIANCA, two types of master files were created per random draw. The first type is the actual training master file that contains the brain extracted and bias corrected T1w and FLAIR images (training set), the FLAIR-to-MNI.mat file, and the manual segmentation mask of the randomly selected (*n* = 10, 15, 20, 25, 30, 35, 40) observations. In each training set, an additional random query subject was added to the training master file, because BIANCA needs an image to predict. The option of BIANCA to save a separate trained model per training set was used. These models contain the hyperparameters of each training procedure and are needed for the prediction of validation data. The second type of master file represents the validation data and comprises all observations that are not in the corresponding training set (internal validation set).

To prevent data leakage, only one observation (BL or FU) of a participant was allowed in each training set. Moreover, if a scan (BL or FU) of a particular subject was selected for the training data, the second scan from the same subject could not be included in the corresponding internal validation set. This resulted in sample sizes of “*n* = 160 - 2 × training sample size” for the internal validation sets. As all internal validation sets included BL and FU images, they were subdivided into a BL and FU internal validation set, so that each participant was included only once per set. The more participants are used for training, the fewer participants are in the internal validation set. We also added 41 images of never trained-on participants to each testing master file to evaluate the performance on an external dataset with a fixed number of participants (external validation set, refer to the “validation sets” in Figure 2 and Tables 1, 2).

### 2.7. Model Training and Prediction

The recommended default settings of BIANCA (Griffanti et al., 2016) were applied. The algorithm was trained with the T1w (in FLAIR space) and FLAIR images as well as the brain mask and MNI152 transformation matrix. The trained models contained the hyperparameters from the training and were saved separately. Each of the 700 saved models (100 random draws per 7 different sample sizes) was used to predict the white matter hyperintensity probability masks on every subject in the validation subsets.

### 2.8. Metric Extraction and Performance Evaluation

From the predicted mask, we extracted white matter hyperintensity volumes at 11 different thresholds (thresholds: 0 to 1 by 0.1) resulting in a total amount of 105,700 predicted 3D images at 11 thresholds = 1,355,200 computations needed for comparison (Table 2).

**Figure 3:**
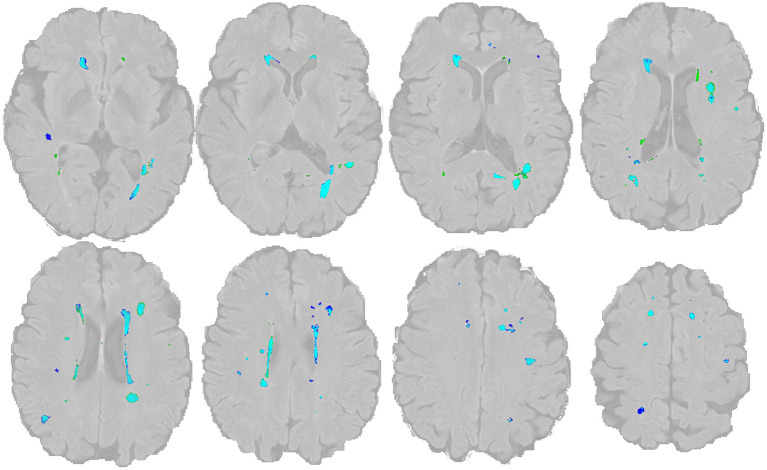
Overlap of WMH lesion masks The manual lesion mask is shown in dark blue, the BIANCA lesion mask is in green (threshold: 0.8). The light blue color indicates the overlap of both. The image and mask are identical to Figure 1.

**Figure 4:**
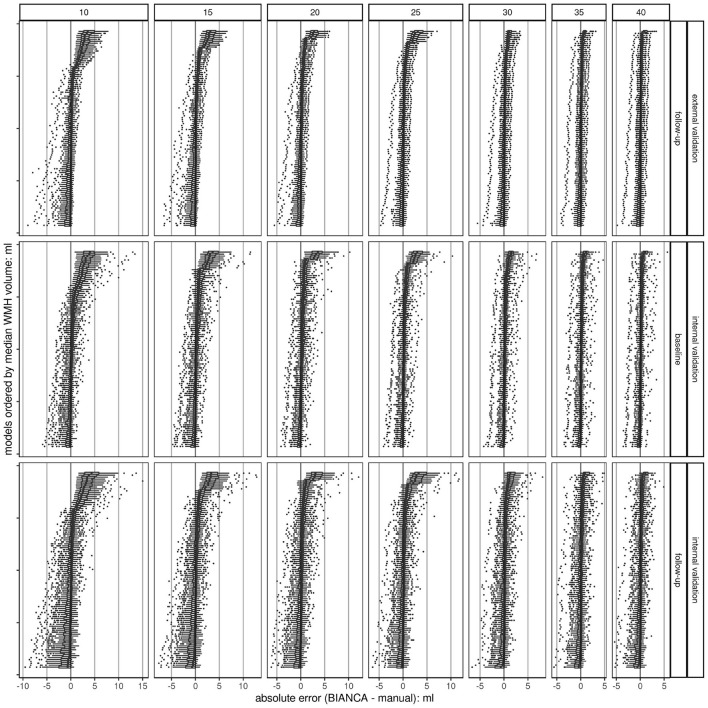
Boxplots of absolute errors (BIANCA predicted volume - manually delineated volume) per trained BIANCA model ordered by median values. Overall, there are 700 models (100 per sample size) at a threshold of 0.8; each dot represents a single observation. The plots are stratified in a grid, horizontally by sample size (*n* = 7) and vertically by validation set (*n* = 3). The higher the sample size, the higher the chance to train a model with a low deviation from the gold standard (smaller range, less outliers, and smaller IQR). This shows a convergence of the accuracy of the models with increased sample size resulting in a more robust performance. The black line indicates the ideal absolute error (BIANCA - manual volume) of 0. Absolute errors greater than 0 show an overestimation of BIANCA, while absolute errors smaller than 0 show an underestimation.

White matter hyperintensity volumes were calculated in ml. The absolute error between the BIANCA predicted volume and the manual (gold standard) volume was calculated, i.e., the deviation (+/-) in ml per model (*n* = 700; 100 random draws of training sets per 7 sample sizes) and participant (Figures 1, 3). Per validation set (internal validation sets at BL and FU and the external validation set), threshold, and model, the mean absolute error was used to calculate mean, median, SD and interquartile range per model and sample size (Figure 4).

**Figure 5:**
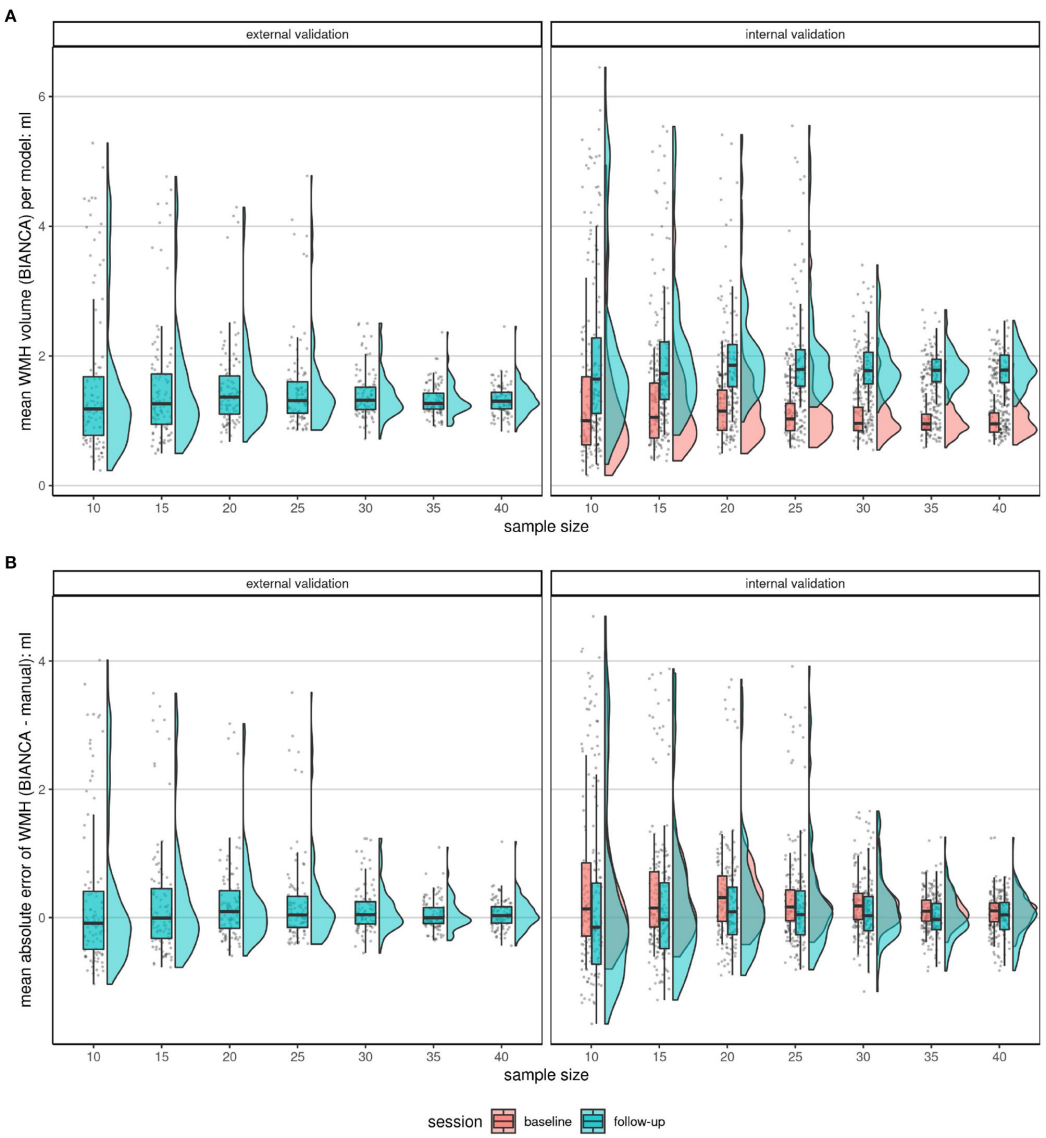
Comparison of the mean BIANCA predicted volume **(A)** and mean absolute errors **(B)** of the two validation set types (internal validation set at BL and FU and external validation set) at increasing sample sizes at a threshold of 0.8. Shown are raincloud plots (Allen et al., 2019) of the mean BIANCA predicted volume **(A)** and the mean absolute error **(B)** by the model (*n* = 100), sample size (*n* = 7), and validation set (*n* = 3). Both figures: The trend shows, that if more subjects were randomly chosen for the training of a BIANCA model, the performance (less outliers, closer IQR) in all sets becomes better. This shows a convergence of performance resulting in a more robust performance. (A): Mean absolute lesion volumes increase from BL to FU. (B): Mean absolute errors are on average larger (more positive) at BL compared to FU. Mean absolute errors greater than 0 point toward an overestimation of white matter hyperintensity volume by the automated segmentation with BIANCA, while mean absolute errors smaller than 0 hint toward an underestimation by BIANCA in comparison with the manual delineation performance (reference standard).

**Figure 6:**
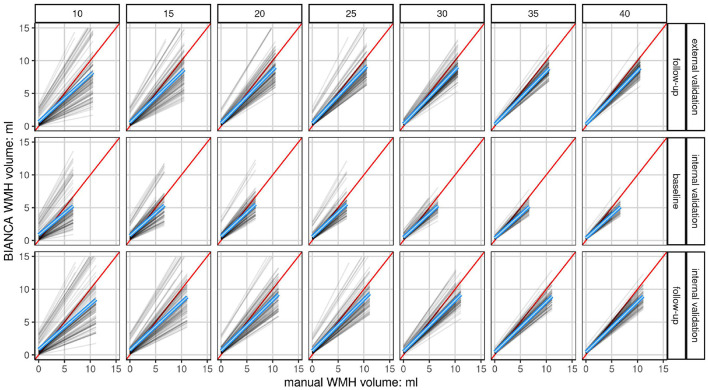
Linear fits of each model show the association of manually segmented total WMH volume to automatically predicted volume (BIANCA) stratified by validation set and sample size. Shown are the linear fits of the manual volume on the x-axis to BIANCA predicted volume on the y-axis of each model (*n* = 100) in a grid stratified by sample size (*n* = 7) horizontally and validation set (*n* = 3) vertically. Only data at a white matter hyperintensity probability threshold of 0.8 is shown. The mean linear fit of all models is indicated by the blue line. The red line indicates the ideal fit, with an intercept of 0 and a slope of 1. The higher the sample size, i.e., the more subjects are drawn from the population, the closer the model performances draw to the mean linear fit of all models. This shows a convergence of the accuracy of each model resulting in a more robust performance. The mean linear fits of all models show an increasing underestimation of white matter hyperintensity volumes by BIANCA with increasing lesion volumes in all sample sizes and sets. Please refer to Supplementary Figure 5 for the same plot showing each participant in a density plot instead of the fit per model.

The ideal threshold was determined by choosing the minimal mean absolute error in the validation sets. At the determined threshold, the mean of means of the models were compared for the three validation sets (internal validation sets at BL and FU, external validation set) using raincloud plots (Figure 5; Allen et al., 2019). These indicate the robustness with increasing sample size. The association of manual segmented volume and algorithm predicted volume per model, sample size, and validation set was visualized with line plots (Figure 6). The underlying dot pattern is visualized with a scatter plot (Supplementary Figure 1). Both of these show the accuracy of each model with increasing sample size. Furthermore, each model is visualized with a separate boxplot (*n* = 700) of absolute errors (BIANCA-manual volume) over these sets shown in Figure 4. We focus on the mean absolute error per model (*n* = 700; 100 random draws of training sets per 7 sample sizes) across the validation sets. The boxplots give insights into the accuracy per model, while all boxplots together indicate robustness. We also visualized the performance with two Bland-Altman like plots (Supplementary Figures 6, 7). Supplementary Figure 6 shows the mean and SD of each model separately by sample size and validation set. Supplementary Figure 7 visualizes the underlying scatter plot. In an additional *post-hoc* analysis, we extracted the proportion of low volume training samples from each training set (< 0.1 ml Supplementary Figure 8, < 0.5 ml Supplementary Figure 9) to investigate, whether there is some systematic effect of the test sample composition on prediction performance. We also evaluated the prediction performance according to lesion volume per observation and sample size (Supplementary Figures 10, 11).

We focused on volume extraction, but additionally calculated the Dice SI to measure the spatial overlap between manually delineated and BIANCA predicted masks (Supplementary Figures 12, 13). The Dice SI is calculated as two times the fraction of voxels in the intersection of the manually delineated and the BIANCA predicted masks divided by the sum of manual mask lesion voxels and BIANCA lesion voxels. Values can be in a range between 0 and 1. High values indicate a good performance, while low values indicate poor performance. The SI was derived from the BIANCA overlap measures tool (Griffanti et al., 2016).

### 2.9. Processing and Hardware

The programming of the processing and analysis was developed in R (version 3.6.2, 2019-12-12, R Core Team, 2019) using RMarkdown (Allaire et al., 2019), Tidyverse (Wickham et al., 2019), and parallelization (Microsoft Corporation and Weston, 2019a,b). All neuroimaging data were converted using dcm2niix (Linux; v1.0.20190902 Li et al., 2016) and saved to brain imaging data structure (BIDS) specification (Gorgolewski et al., 2016) with an in-house built tool (Wulms and Eppe, 2019). The random draws were conducted using the dplyr (Wickham et al., 2019) function *sample_n()* and setting a random seed. All processings were conducted on a Dell ThinkStation-P500 (12 cores x 3.5 GHz, 16 GB Ram) and Ubuntu 18.04 LTS.

## 3. Results

### 3.1. Study Cohort

The sample used in (Table 3) had a mean age of 57 years (SD of 5.7 years) at BL. The right-skewed (manually delineated) white matter hyperintensity volumes (refer to Supplementary Figures 1, 2) at BL had a median of 0.23 ml (IQR of 0.92 ml). At FU the cohort had a mean age of 63 years (SD of 5.7 years) and a median white matter hyperintensity volume of 0.55 ml (IQR of 2.36 ml). When both are pooled together the mean age was 60 years (SD of 6.4 years) and the median white matter hyperintensity volume was 0.34 ml (IQR of 1.6 ml). The external validation subset had a mean age of 60 years (SD of 7.72 years) and a median white matter hyperintensity volume of 0.27 ml (IQR of 1.34 ml). Longitudinal comparisons showed a general intra-individual increase in (manually segmented) WMH volumes over time (Supplementary Figure 1). BIANCA predicted WMH volumes were also greater at FU than at BL (Figure 5).

**Table 3:** Descriptive statistics of the study cohort used for resampling and of the external validation set.

**Study cohort**	**Baseline**	**Follow-up**	**Total (BL+FU)**	**External validation set**
**observations (n)**	**80**	**80**	**160**	**41**
Age (years)	57.5 (5.7); 58.8 (7.3)	63.3 (5.7); 64.6 (7.3)	60.4 (6.4); 60.9 (8.0)	59.5 (7.7); 61.0 (13.9)
Sex (n)				
Women	47 (59%)	47 (59%)	94 (59%)	20 (49%)
Men	33 (41%)	33 (41%)	66 (41%)	21 (51%)
WMH volume^a^ (ml)	0.86 (1.35); 0.23 (0.92)	1.78 (2.57); 0.55 (2.36)	1.32 (2.10); 0.34 (1.60)	1.27 (2.21); 0.27 (1.34)
WMH low volume^a^ observations (n)				
0 ml	12 (15%)	9 (11%)	21 (13%)	7 (17%)
(0 ml, 0.1 ml)	8 (10%)	11 (14%)	18 (11%)	4 (9.8%)
(0.1 ml, 0.5 ml)	10 (12%)	7 (8.8%)	18 (11%)	3 (7.3%)

*The study dataset comprises the resampling dataset that consists of the same 80 participants at BL and FU and the external validation set that consists of additional 41 participants from the FU examination. Statistics presented: continuous variable: mean (SD); median (IQR); categorical variable: n (%)*.

a *based on manual segmentation*.

### 3.2. Threshold Determination

With a mean absolute error of 0.11 ml (SD of 0.26 ml) for BL and 0.01 ml (SD of 0.35 ml) for FU, the threshold of 0.8 to extract white matter hyperintensity volume from the predicted white matter hyperintensity maps showed the minimal deviation from the manual gold standard (Supplementary Figures 3, 4 and Supplementary Tables 1–3). Thus, the threshold of 0.8 was chosen for the following analyses. All mean absolute errors per model, threshold, and sample size are summarized in the Supplementary Figures 3, 4 and Supplementary Tables 1–3.

### 3.3. Comparison of Prediction on Validation Sets

The validation sets were analyzed separately for model performance and visualized using raincloud plots (Allen et al., 2019). The external validation set showed a mean absolute error of 0.31 ml and a standard deviation of 1.2 ml when trained with a random model of 10 images (Figure 5 and Table 4). With increasing training sample size, the SD and interquartile range decreased, while the mean absolute error got closer to 0. For example, a mean absolute error of 0.05 ml (SD of 0.24 ml) resulted from a sample size of 35 images and of 0.06 ml (SD of 0.23 ml) with 40 training samples. The internal validation set at BL had a mean absolute error of 0.11 ml (SD of 0.26 ml) and at FU a mean of 0.01 ml (SD of 0.35 ml). The models trained with 35 or 40 subjects showed less outliers than all other models and indicate a more robust performance of BIANCA.

**Table 4:** Descriptive statistics of the mean absolute errors of lesion volume [Brain Intensity AbNormality Classification Algorithm (BIANCA) predicted white matter hyperintensities (WMH)-manual mask lesion, in ml] per model and validation set at a white matter hyperintensity probability threshold of 0.8.

**Training sets**	**Internal validation sets**		**External validation**
**Sample size**	**Baseline**	**Follow-up**	**Follow-up**
10	0.55 (1.22)	0.28 (1.46)	0.31 (1.20)
15	0.46 (0.93)	0.24 (1.14)	0.26 (0.94)
20	0.40 (0.71)	0.23 (0.83)	0.23 (0.67)
25	0.34 (0.69)	0.24 (0.88)	0.24 (0.72)
30	0.24 (0.40)	0.13 (0.52)	0.13 (0.36)
35	0.13 (0.26)	0.00 (0.36)	0.05 (0.24)
40	0.11 (0.26)	0.01 (0.35)	0.06 (0.23)

*Statistics presented: Mean (SD). For each sample size, 100 random training sets were drawn from the study dataset*.

### 3.4. Association of Manual Segmentation Volume and Predicted Volume

Linear fits of each model (*n* = 700, 100 per sample-size) comparing absolute manual volume vs. BIANCA predicted volume at a threshold of 0.8 are visualized in Figure 6. A scatterplot showing the density of the underlying data is visualized in Supplementary Figure 5. With increasing training sample size, the model performance converges to the mean model performance (blue) indicating more robust predictions. With a lower sample size, the chance to gain an over- or underestimating model, which indicates lower accuracy per model, is increased. Overall, the mean model performance shows that BIANCA generally underestimates white matter hyperintensity volumes.

### 3.5. Mean Absolute Errors per Model, Stratified by Sample-Size and Validation Set

For each model (*n* = 100) a boxplot was created, visualizing the absolute error per observation in the set. These boxplots were then sorted by the median. The higher the sample size, the lower the range of data, and the fewer models are over or underestimating the manual standard (Figure 4). This indicates a higher accuracy per model with an increasing sample size, which results in a more robust performance when randomly choosing training subjects. This can also be observed in the modified Bland-Altman plots (Supplementary Figures 6, 7).

### 3.6. Quality Control

In different intra-subject analyses, we explored whether there are random deviations or systematic effects of lesion volume on the prediction performance (Supplementary Figures 10, 11). Again, in general, the performance converges with increasing sample size. Supplementary Figures 10, 11 also show, that BIANCA seems to underestimate participants with higher lesion volume, whereas participants with lower lesion volumes are more likely to be overestimated. Some random appearing outliers can be observed, regardless of sample size.

The Dice SI shows a poor performance across all sample sizes (Supplementary Figure 12) with a mean SI of 0.14 (SD 0.16) for *n* = 40 training size. The low average spatial overlap is driven by participants with very low lesion volumes (median < 0.2 ml) (Supplementary Figure 13). It can also be observed that the robustness of the prediction increases with higher training sample sizes, which is shown by a smaller range of SI across the models.

### 3.7. Analysis of Training Set Composition

Each training set was *post-hoc* analyzed for the proportion of low volume training samples (< 0.1 ml Supplementary Figure 8, < 0.5 ml Supplementary Figure 9). We could observe for both thresholds (over-all sample sizes and validation sets) that the lower the proportion of low volume training samples, the higher the mean absolute error of the trained model, reflecting an overestimation by BIANCA. Vice versa, in training samples with a high proportion of low volume training samples, BIANCA was more likely to underestimate the WMH volumes. The most accurate performance could be observed for training sets with a 30–40% proportion of low-volume subjects. In general, the performance gets more robust with increasing sample size, which is shown by a smaller range of MAE and smaller IQR.

## 4. Discussion

Seven different effective training sample sizes ranging from 10 to 40 subjects for the training of automated WMH segmentation models with BIANCA were evaluated. Internal and external validation sets were used to compare the automatically estimated lesion volumes with a manual reference standard. The external validation set, with images never used for the training of any model, shows the highest accuracy, defined as the lowest mean absolute error, SD, median, and IQR when trained with 35 and 40 randomly drawn subjects (Figures 4, 5 and Table 4, Supplementary Figures 5, 9). With increasing sample size, the mean absolute error across all models converges to zero, indicating a more robust performance of BIANCA in this population with a very low average lesion load (Supplementary Figures 6, 7). Figure 5B shows differences in the prediction accuracy across study time points. The mean absolute error is on average slightly higher for BL than for FU lesions (Figure 5B). This is most probably due to the combination of a higher proportion of participants with no or low lesion volumes at BL compared to the FU examination, and a general overestimation of small WMH (refer to discussion next paragraph). This should not be confused with the increasing absolute mean lesion volume over time (Figure 5A). This increase in lesion volume is reasonable, regarding the aging cohort. It is also observable, that the predicted mean absolute volumes of the external cohort are on average in between the internal BL and FU data. This also supports a reliable prediction of BIANCA, as the means of the manually delineated volumes of the external cohort (1.27 ml, SD 2.21 ml) were also in between the internal BL (0.86 ml, SD 1.35 ml) and FU volumes (0.86 ml, SD 1.35 ml) (Figure 5 and Table 3).

In the additional intrasubject analyses, we found a strong association between prediction accuracy and lesion volume: the higher the manually delineated lesion volume, the higher the chance for BIANCA to underestimate the lesion volume, while with lower or no manual lesion volumes, BIANCA is more likely to overestimate (Supplementary Figures 10, 11). Moreover, the Dice SI (Supplementary Figures 12, 13) evaluating the spatial overlap between manual and predicted masks was very low, particularly for participants with low or no lesion volume. This inaccurate prediction of small lesion volumes, particularly regarding spatial overlap, has been shown before for other segmentation algorithms (Admiraal-Behloul et al., 2005; Dadar et al., 2017; Heinen et al., 2019; Carass et al., 2020). It might have methodological reasons as well as reasons for true measurement error. The latter concerns the major difficulty of raters and algorithms to correctly identify and delineate single small lesions and contrast them to artifacts or small infarcts (Carass et al., 2020). From a methodological point of view, the Dice SI is particularly dependent on the absolute lesion load and the size of the individual lesions, as a disagreement of only few voxels could lead to a very small SI. Moreover, regarding the direction of measurement error: a volume of zero from a manually delineated mask cannot be underestimated, therefore, any spatial incongruities between manual and predicted mask in subjects with no manually marked lesion lead to an overestimation of the prediction, and a SI of zero, respectively. Furthermore, regarding our own data and analyzes, we neither apply any white matter masks to mask out artifact-prone regions, nor did we use volume thresholds to define a minimal cluster of voxels to be labeled as WMH. This might also support an overestimation of the predicted lesion volumes. It should also be acknowledged that our cohort has a very low proportion of subjects with large WMH, and even these lesions are comparably small to the extensive confluent lesions found in pronounced small vessel disease. Thus, we cannot exclude, that different compositions of training sets, that include subjects with far bigger lesion volumes are generally inferior in the prediction of study samples that comprise small WMH. Nevertheless, we might deduce from Supplementary Figures 8, 9, that a balanced training set, containing low as well as higher lesion subjects, yields the most accurate prediction results, at least regarding volumetric overlap. Therefore, we speculate, that a representative training sample including all the range of possible WMH volumes (also zero) might be optimal. However, as these analyses are *post-hoc*, this hypothesis remains highly speculative.

The limiting factors of our analysis were the availability of training data and computation time. While 201 manually delineated white matter hyperintensity masks, derived from MR sequences acquired with the same scanner, represent a high number in the field of population studies, it is at the lower end of the scale in machine-learning. A higher number of random training sets (e.g., 1,000) would enhance the reliability of our findings, but would take a very long time to delineate manually, and also increase computational time to about a year or longer. Furthermore, with the limited number of 80 subjects to draw from, chances are increased to get duplicate training sets. However, we checked for that, and we were not able to identify an identical set. An associated limitation is the maximum number of 40 training subjects in our analyses. While the accuracy (mean absolute error of the difference between BIANCA prediction and manually delineated masks) of the models does not significantly improve from a training size of about 20–25 onward, we do observe increasing robustness, i.e., a decreasing chance of drawing a deviating model with an increasing number of training participants. Thus, we cannot exclude the possibility, that with a further increase in training sample size the performance, particularly the robustness, would still profit. Nevertheless, in our cohort, a training sample size of (only) 35–40 manually labeled images, which from a cost-benefit view should be realizable in most studies, was adequately robust, i.e., none of the models showed an extreme deviation from the mean fit. Finally, we do not have inter or intrarater agreement measures for our manual delineations. The intention of our manual masks was to gain the most possible validity of WMH masks. While reproducibility will surely be important for the BIANCA algorithm to reliably work in a large cohort, our primary quality goal for the manuals mask was validity. Our way to yield the most valid masks was by consensus decisions, i.e., the harmonization of ratings by constantly having two raters to evaluate each image as well as conducting case-based expert consensus meetings. By design, this maximization of validity was at the cost of potential intra or interrater agreement comparisons.

Brain Intensity AbNormality Classification Algorithm, like other tools, only gives recommendations but does not offer a fully standardized pipeline for image preprocessing. The construction and validation of a pipeline for brain-extraction and bias-correction can be time-consuming. Nevertheless, the impact of preprocessing is important for a valid and reproducible outcome. Accessible, open solutions, beginning with the input of data in a standardized specification format such as BIDS and a containerized environment for preprocessing and analysis (refer to BIDS-Apps Gorgolewski et al., 2017) might help to standardize these approaches (Gorgolewski et al., 2016) in the future. We tested only the recommended default settings for BIANCA and evaluated the influence of the training sample size. Recently, the authors of BIANCA also developed a locally adaptive thresholding method (Sundaresan et al., 2019) to determine the ideal local threshold for the white matter hyperintensity probability maps instead of applying a global threshold. However, this method showed the best improvements in the prediction of WMH when applied to cohorts with higher lesion load.

## 5. Conclusion

Brain Intensity AbNormality Classification Algorithm is a frequently used algorithm for automated white matter hyperintensity segmentation. Our study highlights the importance of choosing a representative training sample of sufficient size for cohorts with low average lesion volumes. This increases the chance of training a model that is close to the ground truth and reflects the lesion properties in the population. However, further work is needed to evaluate the transfer on other cohorts, particularly cohorts comprising very low as well as very high lesion volumes. Further study is also needed to elucidate and ideally improve the inaccurate lesion prediction for small WMH.

## Data Availability Statement

The raw data supporting the conclusions of this article will be made available by the authors, without undue reservation.

## Ethics Statement

The studies involving human participants were reviewed and approved by Ethics Committee of the University of Muenster and the Westphalian Chamber of Physicians in Muenster, Germany. The patients/participants provided their written informed consent to participate in this study. Written informed consent was obtained from the individual(s) for the publication of any potentially identifiable images or data included in this article.

## Author Contributions

NW drafted the manuscript, conducted and programmed all preprocessing, statistical analysis, and reproducible workflow. CH and LR segmented all white matter hyperintensities (WMH), prepared the manual masks used for training and testing, and reviewed the manuscript. NB advised and reviewed the statistical methods of the manuscript. BS and HM supervised the analyses, trained CH and LR in white matter hyperintensity delineation, and together with KB (principal investigator of the BiDirect Study) helped substantially in writing and editing of the manuscript. All authors contributed to the article and approved the submitted version.

## Funding

The BiDirect Study is funded by the Federal Ministry of Education and Research, Germany (grant nos. #01ER0816, #01ER1205, and #01ER1506).

## Conflict of Interest

The authors declare that the research was conducted in the absence of any commercial or financial relationships that could be construed as a potential conflict of interest.

## Publisher's Note

All claims expressed in this article are solely those of the authors and do not necessarily represent those of their affiliated organizations, or those of the publisher, the editors and the reviewers. Any product that may be evaluated in this article, or claim that may be made by its manufacturer, is not guaranteed or endorsed by the publisher.
