# Supplementary material for: The Effect of Training Sample Size on the Prediction of White Matter Hyperintensity Volume in a Healthy Population Using BIANCA
Source: Front Aging Neurosci. 2022 Jan 11;13:720636. doi: 10.3389/fnagi.2021.720636 (PMC8812526; doi:10.3389/fnagi.2021.720636)
Supplement: Supplementary file 1 [file Data_Sheet_1.pdf]

Supplementary Material

0.1 Tables

The following tables S1 to S3 were created by calculating the mean absolute error of BIANCA predicted mask and manual mask per participant in ml. These mean absolute errors were then pooled for each model (n = 100) per sample size (n = 7) and threshold (n = 11). The mean absolute errors in each model were then used, to calculate mean, standard deviation, median and interquartile range over the 100 models per sample size and threshold.

Table S1. Threshold determination by using the external validation set: mean absolute error per model (n = 100) at each threshold (11) and sample size (7) was calculated.

Threshold	10, n =100	15, n = 100	20, n = 100	25, n = 100	30, n = 100	35, n = 100	40, n = 100
0	14 +- 15; 8; 8	12 +- 12; 8; 6	11 +- 7; 9; 5	11 +- 8; 9; 5	10 +- 3; 9; 3	9 +- 2; 9; 2	9 +- 2; 9; 2
0.1	7.6 +- 8.7; 4.2; 3.8	6.5 +- 6.7; 4.3; 3.5	5.7 +- 4.1; 4.5; 2.5	5.7 +- 4.6; 4.2; 2.4	4.8 +- 1.8; 4.3; 1.7	4.4 +- 1.1; 4.3; 1.3	4.4 +- 1.2; 4.2; 1.2
0.2	4.69 +- 5.56; 2.55; 2.46	4.00 +- 4.28; 2.55; 2.30	3.50 +- 2.63; 2.77; 1.67	3.52 +- 3.02; 2.65; 1.54	2.95 +- 1.18; 2.58; 1.24	2.66 +- 0.76; 2.52; 0.86	2.67 +- 0.77; 2.56; 0.85
0.3	3.24 +- 3.99; 1.72; 1.94	2.78 +- 3.07; 1.75; 1.64	2.45 +- 1.94; 1.94; 1.31	2.47 +- 2.21; 1.82; 1.19	2.06 +- 0.89; 1.81; 0.93	1.84 +- 0.58; 1.71; 0.69	1.85 +- 0.58; 1.77; 0.66
0.4	2.32 +- 3.03; 1.21; 1.54	2.02 +- 2.35; 1.22; 1.39	1.79 +- 1.52; 1.37; 1.06	1.80 +- 1.72; 1.31; 0.98	1.50 +- 0.72; 1.30; 0.75	1.32 +- 0.47; 1.23; 0.57	1.33 +- 0.47; 1.26; 0.55
0.5	1.67 +- 2.40; 0.80; 1.30	1.46 +- 1.87; 0.82; 1.21	1.31 +- 1.24; 0.98; 0.92	1.32 +- 1.39; 0.92; 0.81	1.08 +- 0.60; 0.92; 0.63	0.93 +- 0.40; 0.85; 0.47	0.94 +- 0.39; 0.89; 0.47
0.6	1.15 +- 1.92; 0.50; 1.19	1.01 +- 1.50; 0.51; 1.07	0.91 +- 1.02; 0.68; 0.80	0.93 +- 1.13; 0.60; 0.69	0.74 +- 0.51; 0.61; 0.54	0.61 +- 0.34; 0.53; 0.39	0.62 +- 0.32; 0.59; 0.39
0.7	0.72 +- 1.53; 0.21; 1.04	0.62 +- 1.20; 0.23; 0.93	0.57 +- 0.83; 0.36; 0.66	0.58 +- 0.92; 0.32; 0.58	0.43 +- 0.44; 0.34; 0.45	0.33 +- 0.29; 0.26; 0.31	0.33 +- 0.27; 0.31; 0.32
0.8	0.31 +- 1.20; -0.09; 0.90	0.26 +- 0.94; -0.01; 0.78	0.23 +- 0.67; 0.09; 0.59	0.24 +- 0.72; 0.04; 0.48	0.13 +- 0.36; 0.05; 0.35	0.05 +- 0.24; 0.00; 0.25	0.06 +- 0.23; 0.03; 0.26
0.9	-0.11 +- 0.86; -0.39; 0.77	-0.13 +- 0.68; -0.30; 0.61	-0.13 +- 0.50; -0.22; 0.47	-0.13 +- 0.52; -0.25; 0.37	-0.19 +- 0.29; -0.27; 0.29	-0.26 +- 0.19; -0.30; 0.22	-0.25 +- 0.19; -0.27; 0.20
1	-0.73 +- 0.35; -0.81; 0.43	-0.70 +- 0.30; -0.74; 0.40	-0.69 +- 0.23; -0.73; 0.28	-0.69 +- 0.22; -0.72; 0.22	-0.70 +- 0.17; -0.73; 0.18	-0.73 +- 0.12; -0.74; 0.14	-0.73 +- 0.11; -0.74; 0.11

[1] Statistics presented: Mean +- SD; Median; IQR

Table S2. Threshold determination by using the internal validation set (baseline): mean absolute error per model (n = 100) at each threshold (11) and sample size (7) was calculated.

Threshold	10, n = 100	15, n = 100	20, n = 100	25, n = 100	30, n = 100	35, n = 100	40, n = 100
0	13 +- 13; 8; 6	11 +- 10; 8; 6	10 +- 6; 8; 4	10 +- 7; 8; 4	9 +- 3; 8; 3	8 +- 2; 8; 2	8 +- 2; 8; 2
0.1	7.2 +- 7.7; 4.2; 3.5	6.2 +- 5.8; 4.2; 3.4	5.4 +- 3.7; 4.5; 2.6	5.3 +- 4.0; 4.2; 2.4	4.5 +- 1.7; 4.0; 1.5	4.1 +- 1.1; 3.9; 1.3	4.0 +- 1.1; 3.8; 1.4
0.2	4.76 +- 5.13; 2.86; 2.68	4.04 +- 3.83; 2.81; 2.56	3.51 +- 2.50; 3.03; 1.94	3.44 +- 2.73; 2.71; 1.46	2.88 +- 1.20; 2.56; 1.05	2.58 +- 0.77; 2.50; 1.02	2.56 +- 0.80; 2.44; 1.00
0.3	3.47 +- 3.83; 2.11; 2.28	2.96 +- 2.85; 2.09; 2.09	2.58 +- 1.93; 2.21; 1.65	2.51 +- 2.07; 1.95; 1.16	2.10 +- 0.95; 1.84; 0.88	1.85 +- 0.63; 1.77; 0.78	1.83 +- 0.64; 1.74; 0.81
0.4	2.62 +- 3.02; 1.59; 1.98	2.25 +- 2.26; 1.57; 1.77	1.96 +- 1.57; 1.71; 1.43	1.90 +- 1.66; 1.45; 0.93	1.57 +- 0.80; 1.37; 0.75	1.36 +- 0.53; 1.28; 0.62	1.35 +- 0.53; 1.28; 0.69
0.5	1.98 +- 2.44; 1.17; 1.76	1.71 +- 1.84; 1.16; 1.50	1.50 +- 1.31; 1.33; 1.19	1.44 +- 1.36; 1.07; 0.77	1.17 +- 0.68; 1.01; 0.64	0.99 +- 0.46; 0.92; 0.53	0.98 +- 0.45; 0.93; 0.57
0.6	1.46 +- 1.98; 0.79; 1.57	1.26 +- 1.50; 0.83; 1.25	1.10 +- 1.09; 0.97; 1.01	1.05 +- 1.12; 0.74; 0.68	0.84 +- 0.58; 0.74; 0.58	0.68 +- 0.39; 0.61; 0.47	0.66 +- 0.38; 0.64; 0.46
0.7	0.99 +- 1.59; 0.47; 1.38	0.85 +- 1.21; 0.48; 1.06	0.75 +- 0.90; 0.63; 0.88	0.69 +- 0.90; 0.44; 0.60	0.53 +- 0.49; 0.45; 0.50	0.40 +- 0.33; 0.35; 0.41	0.38 +- 0.31; 0.36; 0.38
0.8	0.55 +- 1.22; 0.14; 1.14	0.46 +- 0.93; 0.15; 0.86	0.40 +- 0.71; 0.31; 0.70	0.34 +- 0.69; 0.16; 0.48	0.24 +- 0.40; 0.18; 0.41	0.13 +- 0.26; 0.10; 0.33	0.11 +- 0.26; 0.10; 0.29
0.9	0.10 +- 0.83; -0.19; 0.81	0.05 +- 0.65; -0.16; 0.65	0.02 +- 0.51; -0.05; 0.46	-0.02 +- 0.48; -0.13; 0.38	-0.07 +- 0.30; -0.12; 0.32	-0.16 +- 0.20; -0.17; 0.24	-0.17 +- 0.21; -0.16; 0.23
1	-0.50 +- 0.30; -0.56; 0.37	-0.48 +- 0.27; -0.53; 0.31	-0.48 +- 0.22; -0.50; 0.25	-0.49 +- 0.20; -0.53; 0.22	-0.49 +- 0.18; -0.49; 0.19	-0.54 +- 0.15; -0.54; 0.19	-0.55 +- 0.17; -0.53; 0.17

[1] Statistics presented: Mean +- SD; Median; IQR

Table S3. Threshold determination by using the internal validation set (follow-up): mean absolute error per model (n = 100) at each threshold (11) and sample size (7) was calculated.

Threshold	10, n = 100	15, n = 100	20, n = 100	25, n = 100	30, n = 100	35, n = 100	40, n = 100
0	14 +- 15; 9; 7	13 +- 11; 9; 6	12 +- 7; 10; 5	12 +- 8; 9; 5	11 +- 4; 9; 3	10 +- 2; 9; 3	10 +- 2; 9; 2
0.1	8.0 +- 8.7; 4.7; 3.8	6.9 +- 6.6; 4.9; 3.6	6.2 +- 4.1; 5.0; 2.5	6.2 +- 4.6; 4.8; 2.4	5.4 +- 1.9; 4.8; 2.0	4.9 +- 1.2; 4.7; 1.4	4.9 +- 1.2; 4.7; 1.3
0.2	5.10 +- 5.71; 2.94; 2.73	4.41 +- 4.29; 3.07; 2.43	3.96 +- 2.73; 3.29; 1.76	3.96 +- 3.11; 3.08; 1.64	3.40 +- 1.31; 3.02; 1.20	3.10 +- 0.79; 2.88; 0.94	3.08 +- 0.79; 2.97; 0.93
0.3	3.59 +- 4.19; 2.05; 2.19	3.13 +- 3.16; 2.14; 1.92	2.84 +- 2.06; 2.31; 1.47	2.84 +- 2.34; 2.25; 1.31	2.43 +- 1.02; 2.12; 0.96	2.19 +- 0.63; 2.04; 0.78	2.18 +- 0.62; 2.10; 0.80
0.4	2.60 +- 3.27; 1.48; 1.95	2.30 +- 2.48; 1.54; 1.64	2.10 +- 1.66; 1.67; 1.21	2.11 +- 1.86; 1.66; 1.17	1.79 +- 0.86; 1.53; 0.80	1.60 +- 0.53; 1.48; 0.66	1.59 +- 0.52; 1.55; 0.71
0.5	1.88 +- 2.65; 0.97; 1.75	1.67 +- 2.03; 1.06; 1.48	1.55 +- 1.39; 1.23; 1.08	1.56 +- 1.54; 1.20; 1.00	1.31 +- 0.74; 1.11; 0.71	1.13 +- 0.47; 1.04; 0.59	1.13 +- 0.46; 1.10; 0.61
0.6	1.29 +- 2.19; 0.57; 1.60	1.16 +- 1.68; 0.67; 1.32	1.08 +- 1.17; 0.83; 0.98	1.09 +- 1.29; 0.80; 0.87	0.89 +- 0.66; 0.73; 0.66	0.74 +- 0.43; 0.67; 0.55	0.74 +- 0.41; 0.73; 0.52
0.7	0.77 +- 1.81; 0.22; 1.44	0.69 +- 1.40; 0.31; 1.18	0.65 +- 1.00; 0.46; 0.87	0.66 +- 1.08; 0.44; 0.79	0.51 +- 0.58; 0.37; 0.61	0.37 +- 0.39; 0.30; 0.49	0.37 +- 0.38; 0.39; 0.49
0.8	0.28 +- 1.46; -0.15; 1.26	0.24 +- 1.14; -0.03; 1.03	0.23 +- 0.83; 0.09; 0.74	0.24 +- 0.88; 0.05; 0.69	0.13 +- 0.52; 0.03; 0.53	0.00 +- 0.36; -0.03; 0.41	0.01 +- 0.35; 0.04; 0.42
0.9	-0.26 +- 1.10; -0.56; 1.12	-0.26 +- 0.88; -0.45; 0.90	-0.25 +- 0.66; -0.37; 0.64	-0.25 +- 0.68; -0.38; 0.57	-0.31 +- 0.45; -0.35; 0.50	-0.42 +- 0.33; -0.44; 0.37	-0.41 +- 0.33; -0.37; 0.37
1	-1.08 +- 0.51; -1.17; 0.69	-1.02 +- 0.45; -1.08; 0.63	-1.00 +- 0.36; -0.99; 0.48	-1.01 +- 0.35; -1.05; 0.39	-1.00 +- 0.33; -1.02; 0.38	-1.08 +- 0.28; -1.06; 0.37	-1.08 +- 0.31; -1.04; 0.38

[1] Statistics presented: Mean +- SD; Median; IQR

0.2 Figures

0.2.1 Figures - cohort visualization

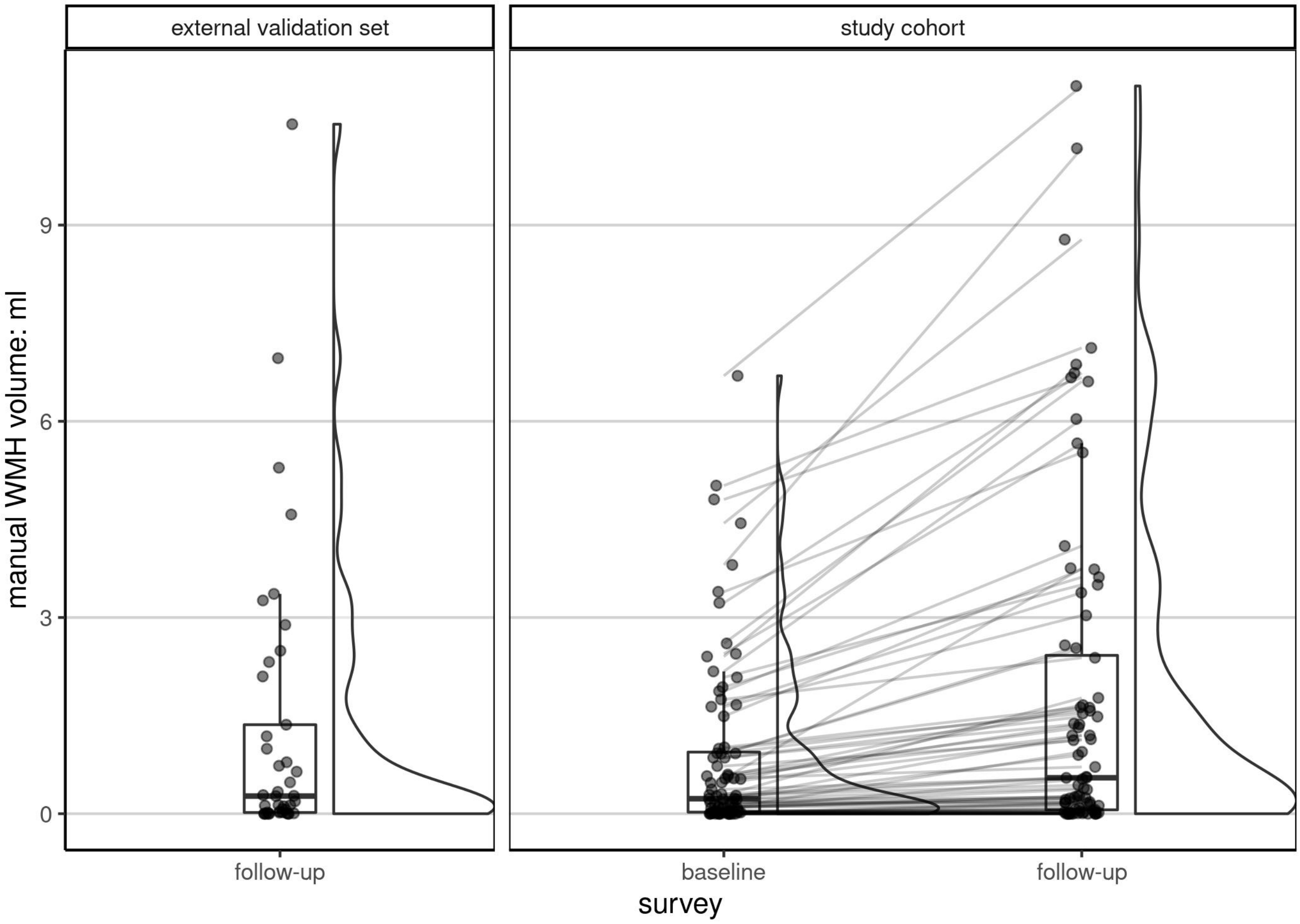

Figure S1. Raincloudplot (Allen et al., 2019) of the study cohort (used for resampling, per baseline and follow-up) and the external validation set Each dot represents the manually segmented lesion volume in ml of a participant in the cohort and survey. Lines are connecting the baseline and follow-up observation of the same participant.

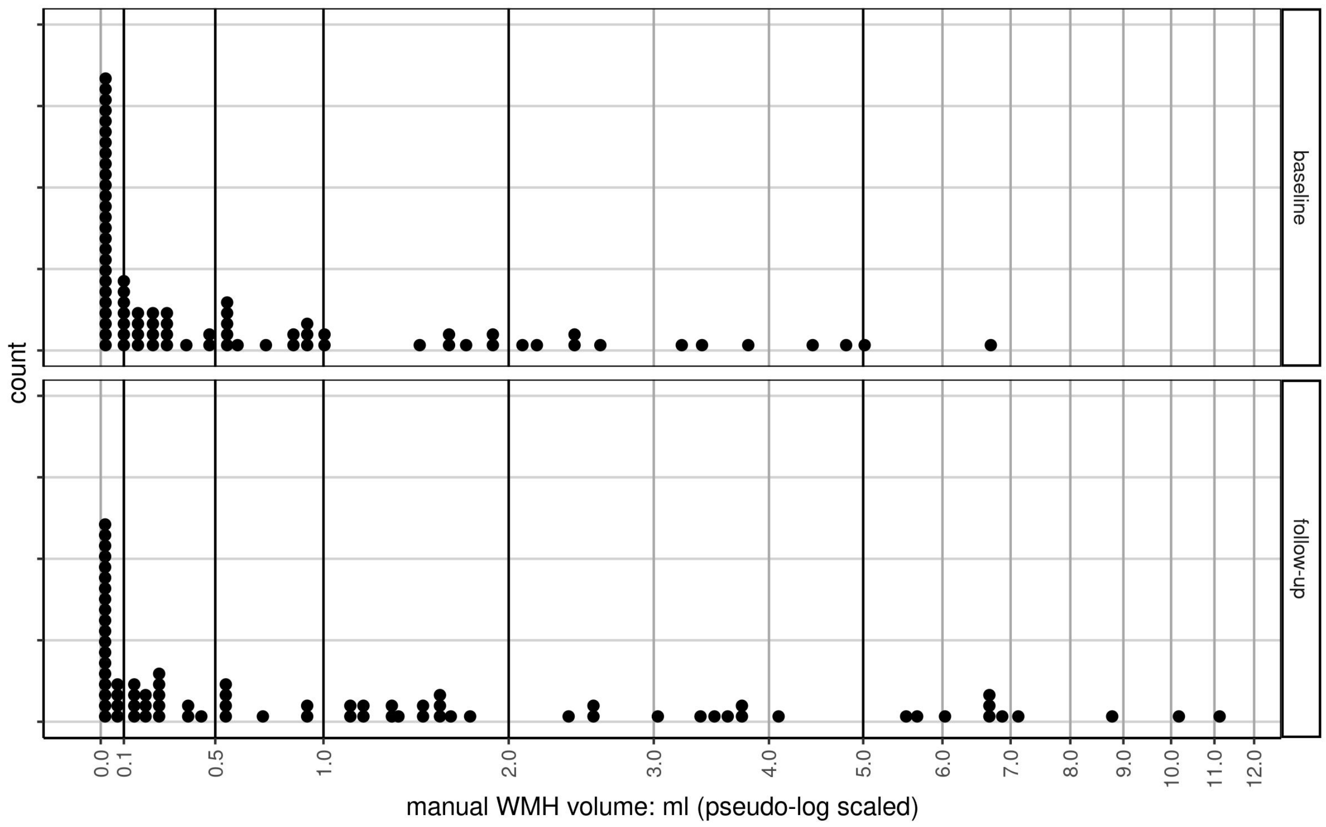

Figure S2. Dotplot of the distribution of the study dataset, which was used for resampling Each dot represents a subject. The lesion volume is the determined manual volume. The x-scale is pseudo-log10-transformed.

0.2.2 Threshold determination

The lesion probability threshold is determined after the prediction. The following boxplots visualize the data from tables S1 to S3 and should visualize how the threshold was chosen that was used for the analysis in the main manuscript.

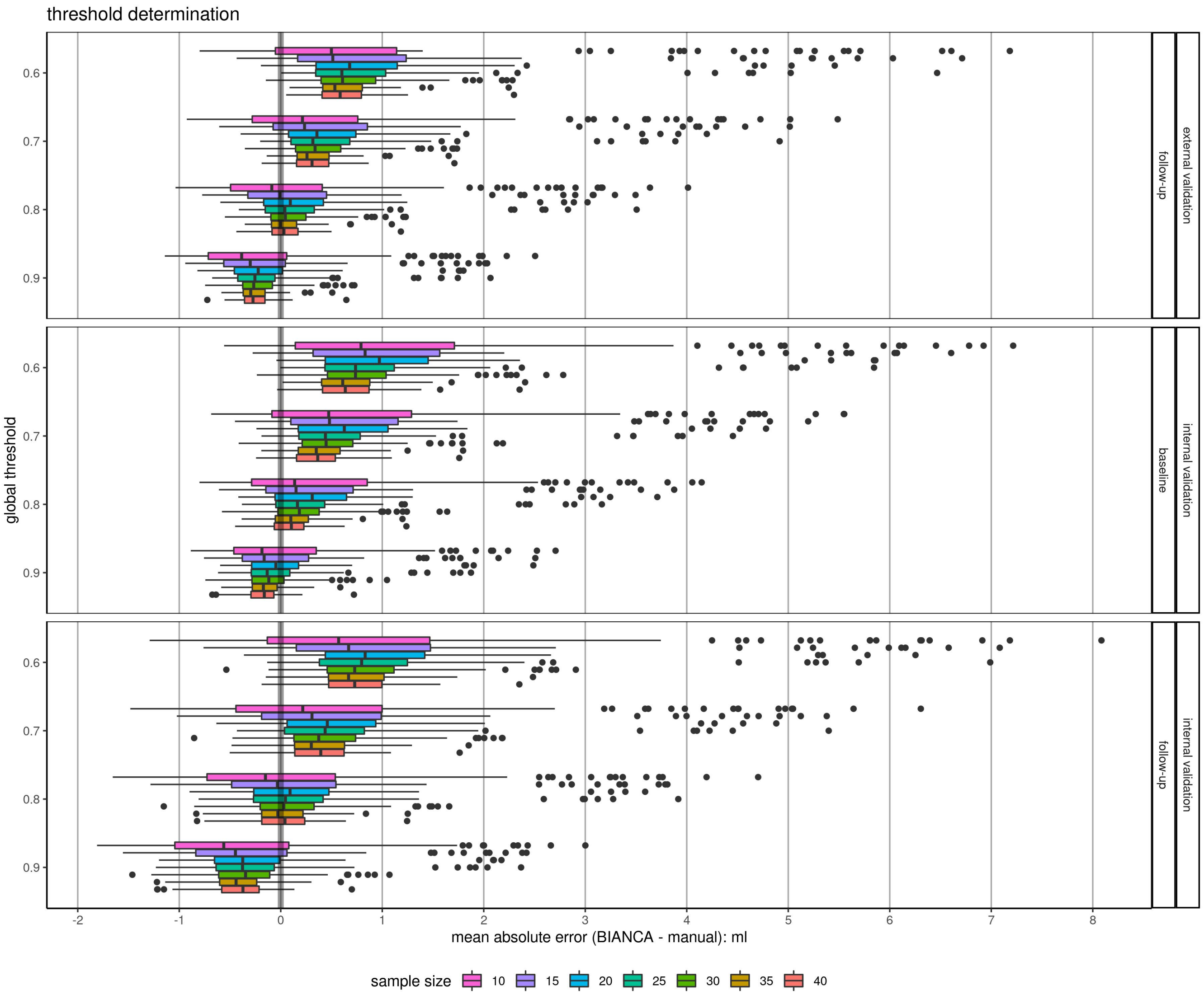

Figure S3. BIANCA threshold determination Boxplots of the mean absolute errors of each model (n = 100) per sample size (n = 7) and validation set (n = 3) are visualized. Each dot represents the mean absolute error of one model. Thresholds below 0.6 were excluded to show the deviations in more detail. A threshold of 0.8 shows the minimal volume deviation from the manual gold standard (black line) in the internal validation set and external validation cohort. The black line indicates the ideal volume difference (BIANCA - manual volume) of 0.

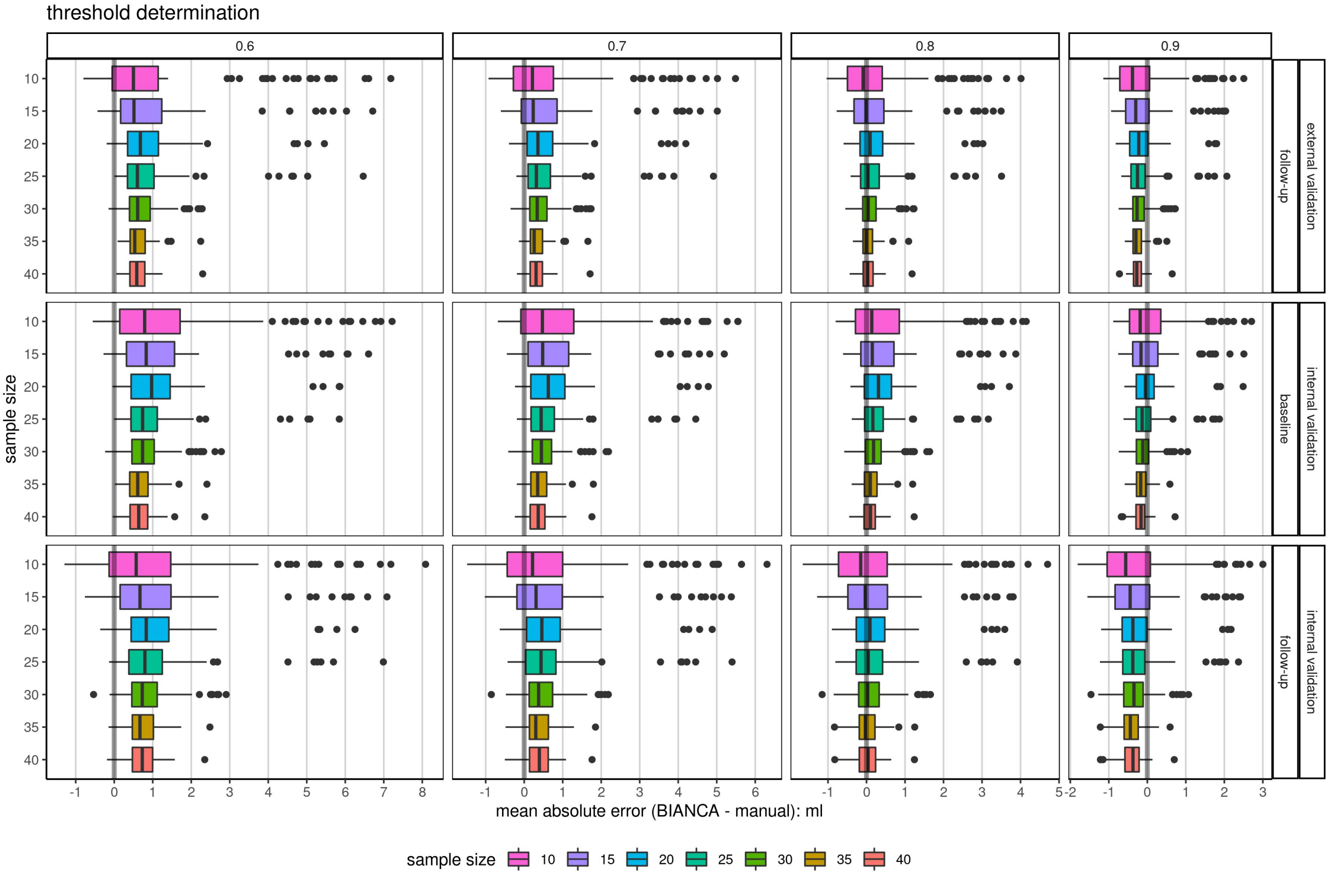

Figure S4. BIANCA threshold determination. Boxplots of the mean absolute errors of each model (n = 100), sample size (n = 7) and validation set (n = 3). Each dot represents the mean absolute error of one model. Thresholds below 0.6 were excluded to show the performance in more detail. The linear trend is comparable across all sample sizes and validation sets. A threshold of 0.8 shows the minimal volume deviation from the manual gold standard (black line) in the internal validation set and external validation cohort. The black line indicates the ideal volume difference (BIANCA - manual volume) of 0.

0.2.3 Visualization of each subject per sample size, validation set and sample size

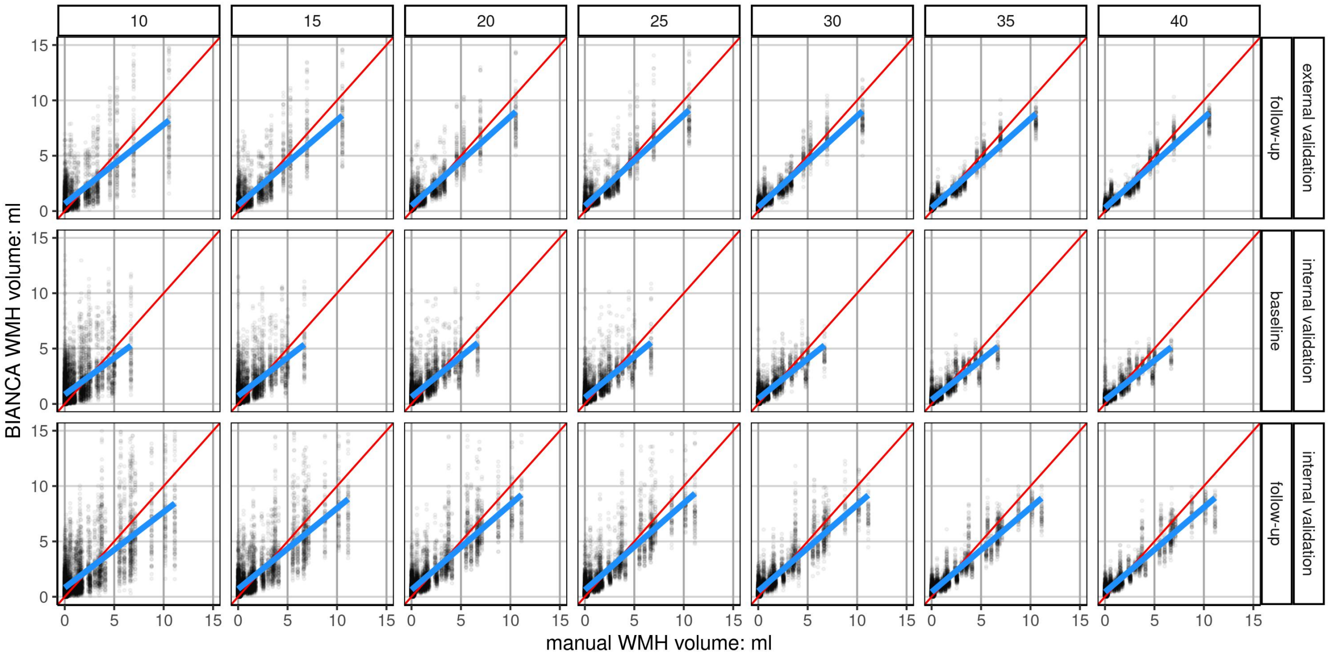

Figure S5. Scatter plots showing the association of manual volume with BIANCA predicted volume Each dot represents a predicted mask from one model. Please note, that dots are translucent to show density effects. The blue line is the linear fit of all predicted masks fitting the manual volume to BIANCA predicted volume. The red line indicates the ideal line, with an intercept of 0 and a slope of 1. The plot is stratified in a grid by sample size (n = 7) horizontally and validation set (n= 3) vertically. All predicted masks were thresholded with 0.8. The more subjects are drawn randomly for training, the closer the general model performance indicating a more robust prediction. The accuracy derived from the fit points towards an increasing underestimation with increasing lesion volumes.

0.2.4 Bland-Altman like plots

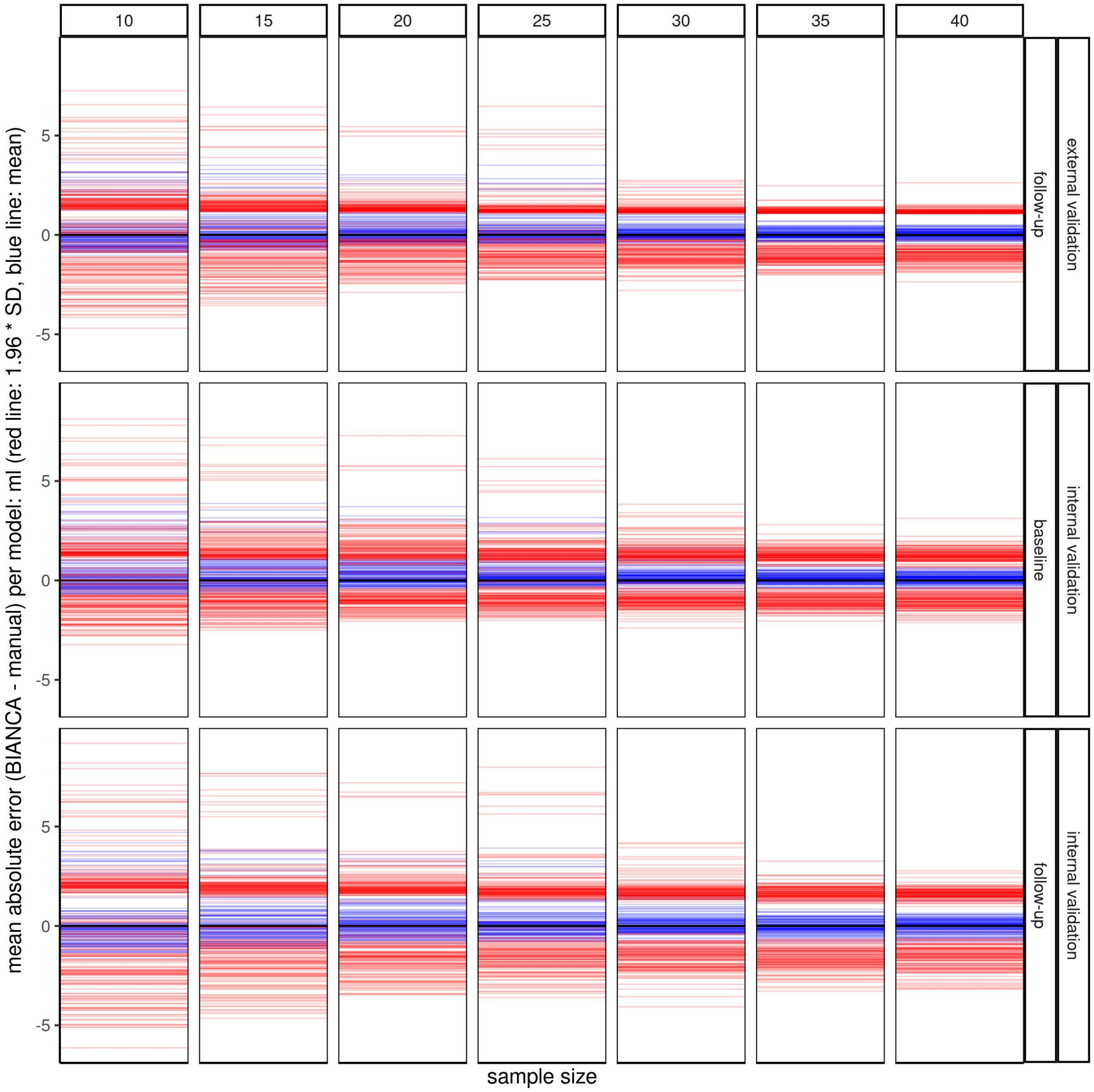

Figure S6. Bland-Altman like plots of each model at threshold of 0.8 Shown are only the SD and mean of each model (n = 100), sample size (n = 7) and validation set (n = 3). The more subjects are drawn randomly for training, the closer the general model performance, and the smaller chance of drawing a deviating model. The blue line indicates the mean of each model. The red line indicates the 1.96 standard deviation. The randomly drawn models trained with 35 or 40 images show a more robust performance than the models, which were trained with 10 to 25 images.

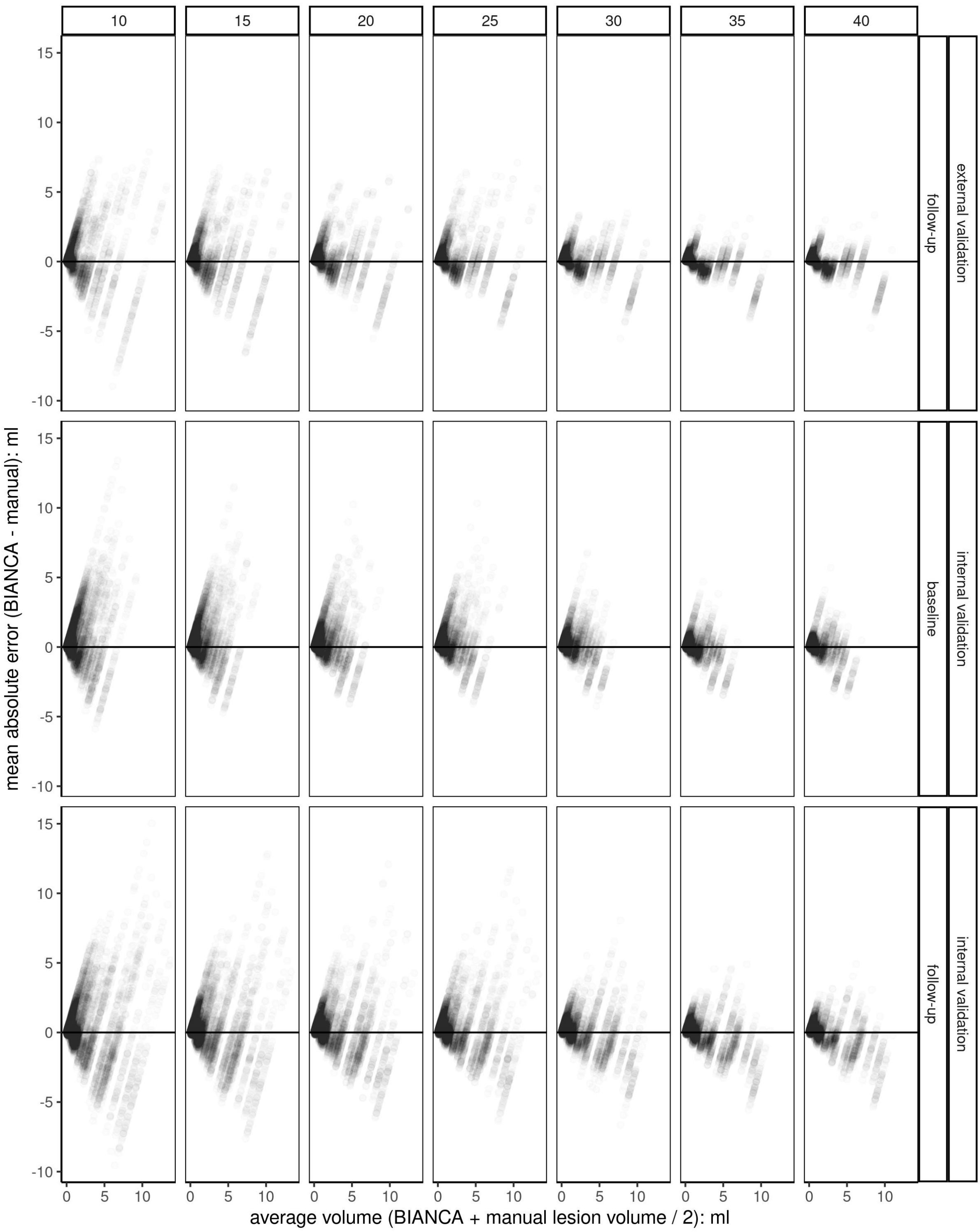

Figure S7. Bland-Altman like plots of each predicted mask at threshold of 0.8 Shown are the mean absolute error of each predicted mask and manual segmentation by average volume of both volumes stratified by sample size (n = 7) and validation set (n = 3). Each dot represents the mean absolute error of a predicted mask. The more subjects are drawn randomly for training, the closer the general model performance, and the smaller the chance of drawing a deviating model. The black lines shows an ideal performance. The randomly drawn models trained with 35 or 40 images show a more robust performance than the models, which were trained with 10 to 25 images.

0.2.5 Internal validation set effects

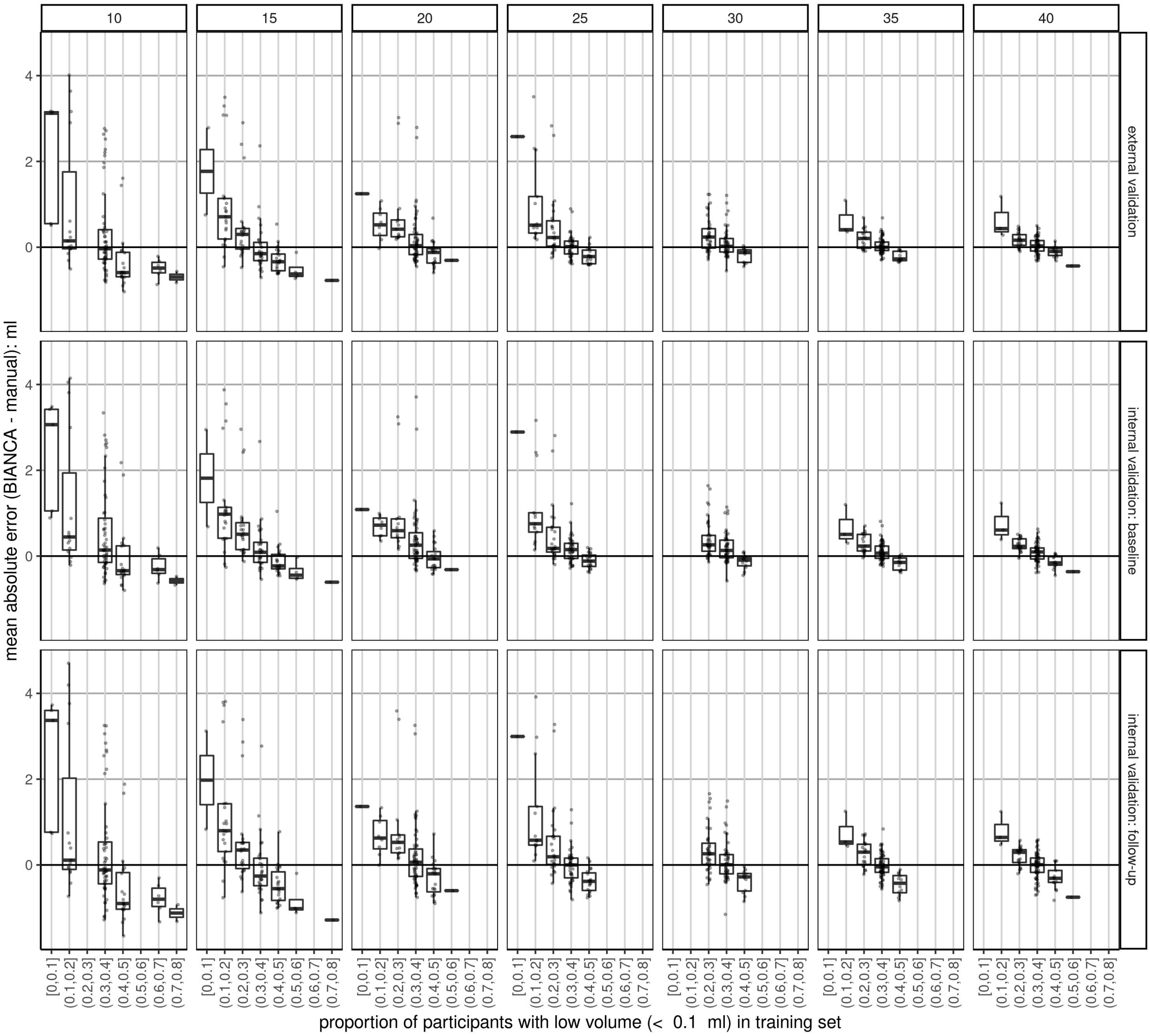

Figure S8. Comparison of different proportions of participants with very low lesion volumes (binarized at threshold of 0.1 ml) in the training sets at increasing sample sizes at a threshold of 0.8. Shown are boxplots of the mean absolute error per model (n = 100), sample size (n = 7) and validation set (n = 3). The trend shows, that the more subjects with very low lesion volume (< 0.1 ml) were randomly drawn for training of a model, the lower the chance of BIANCA to overestimate in all sets. Mean absolute errors greater than 0 point towards an overestimation of white matter hyperintensity volume with BIANCA, while mean absolute errors smaller than 0 hint towards an underestimation of BIANCA in comparison with the manual delineation performance.

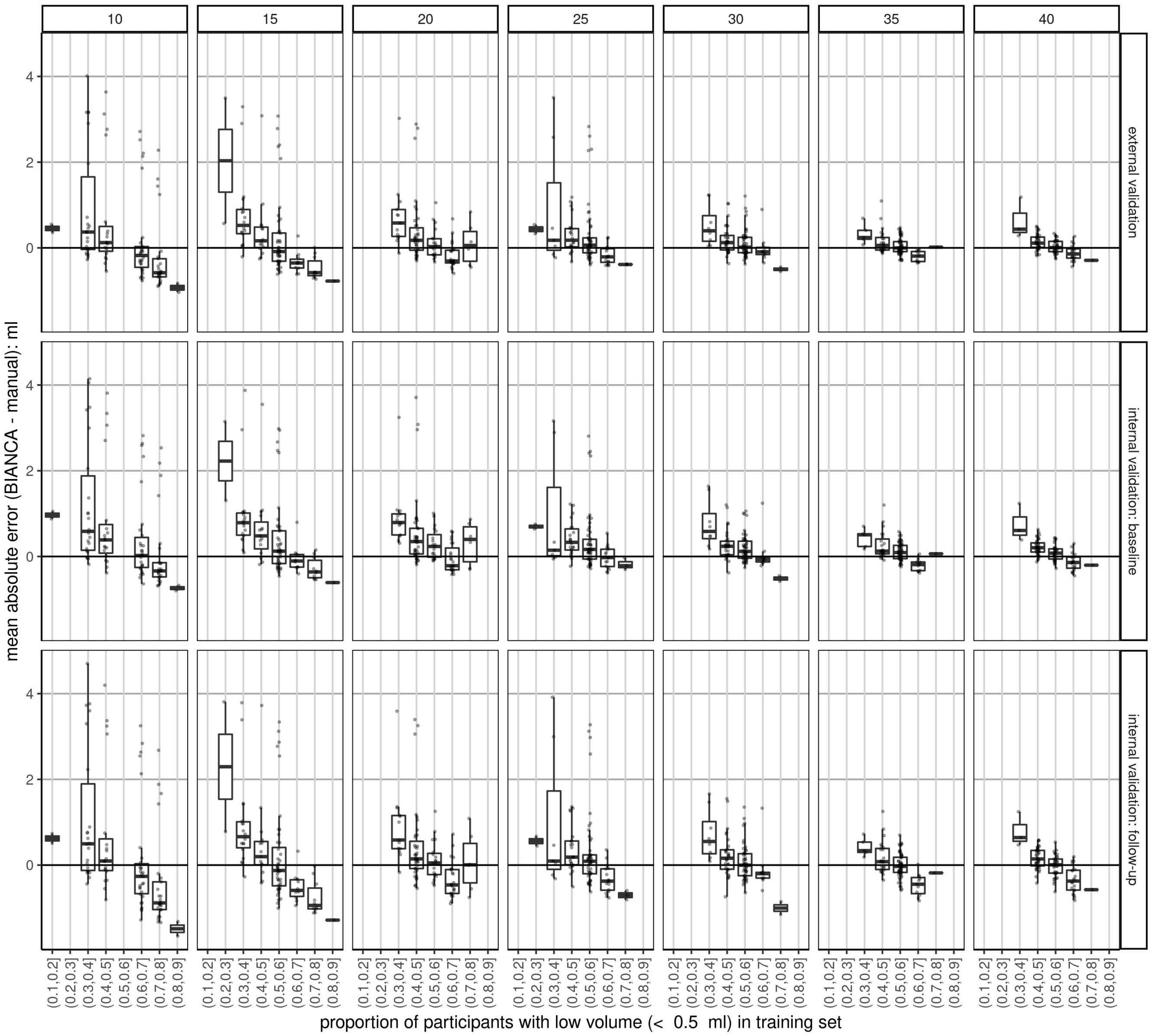

Figure S9. Comparison of different proportions of participants with low WMH volume (binarized at threshold of 0.5 ml) in the training sets at increasing sample sizes at a threshold of 0.8. Shown are boxplots of the mean absolute error per model (n = 100), sample size (n = 7) and validation set (n = 3). These values are derived by thresholding the white matter hyperintensity probability at 0.8. The trend shows, that the more subjects with low lesion volume (< 0.5 ml) were randomly drawn for training of a model, the lower the chance of BIANCA to overestimate in all sets. Mean absolute errors greater than 0 point towards an overestimation of white matter hyperintensity volume with BIANCA, while mean absolute errors smaller than 0 hint towards an underestimation of BIANCA in comparison with the manual delineation.

0.2.6 Intra-subject effects

The intra-subject analyses explore random deviations of the different model predictions as well as potential systematic effects of lesion volume. They show the intra-subject range of the mean absolute error over all models stratified by sample size and validation set, sorted by manual lesion volume (figures S10, S11). Each participant is visualized by a boxplot.

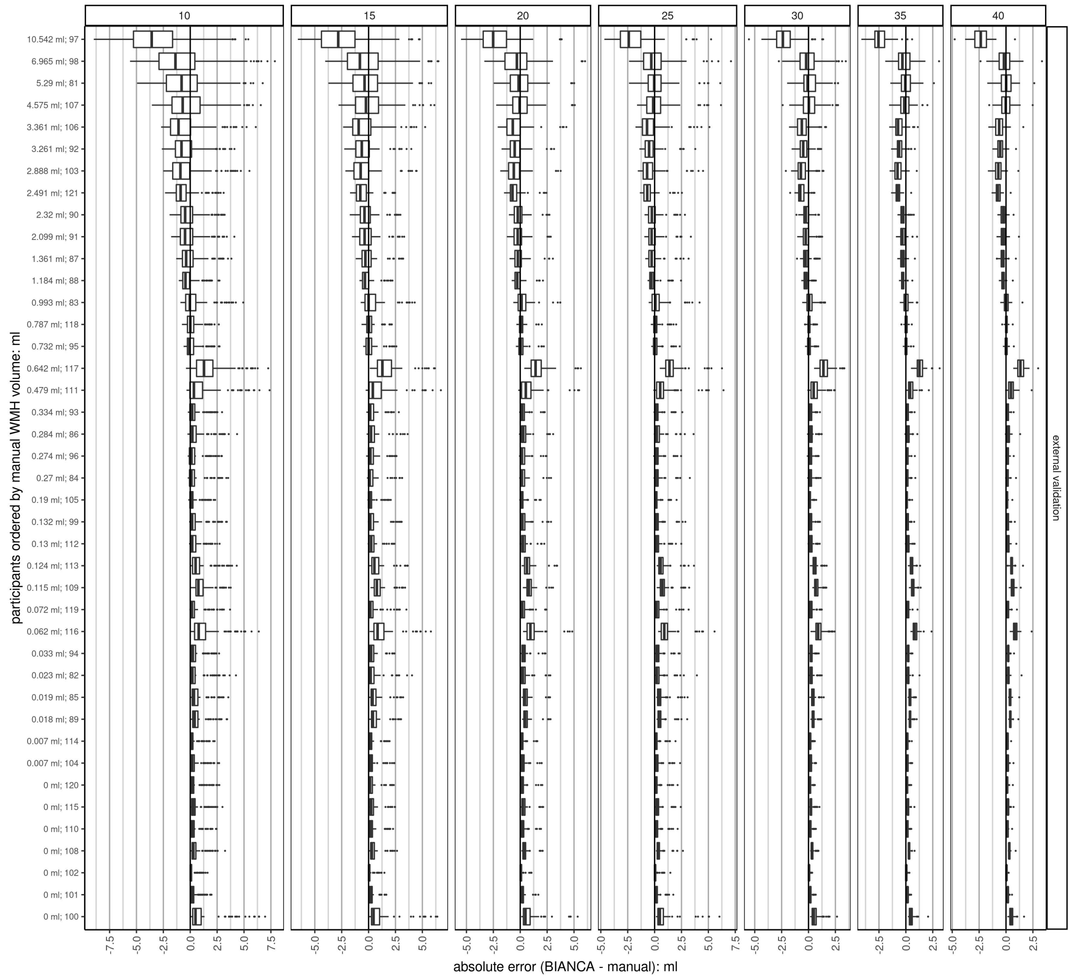

Figure S10. Intra-subject effects (external validation), sorted by manual lesion volume: Boxplots of each subject (n = 41) in the external validation, stratified by sample size (n = 7) and sorted by manual lesion volume at threshold 0.8. Shown are boxplots for each participant of the absolute error of all models. Each dot is a single observation. The black line indicates the ideal absolute error (BIANCA - manual volume) of 0 ml. Absolute errors greater than 0 ml show an overestimation of BIANCA, while absolute errors smaller than 0 ml show an underestimation. The figure shows two main systematic effects: (1) The higher the sample size, the higher the chance to train a model with a low deviation from the gold standard (smaller range of outliers and IQR), indicating a more robust prediction. (2) Regarding accuracy: The higher the lesion volume, the more likely BIANCA underestimates the lesion volume. Moreover, mean absolute errors of some (random) observations deviate from the cohort throughout all sample sizes. This could hint towards effects of a certain anatomy, that lead to a general under- or overestimation by BIANCA. Please note: The scale of the x-axis is the same across sample sizes, but the x-axis is truncated to cover only the area of existing values.

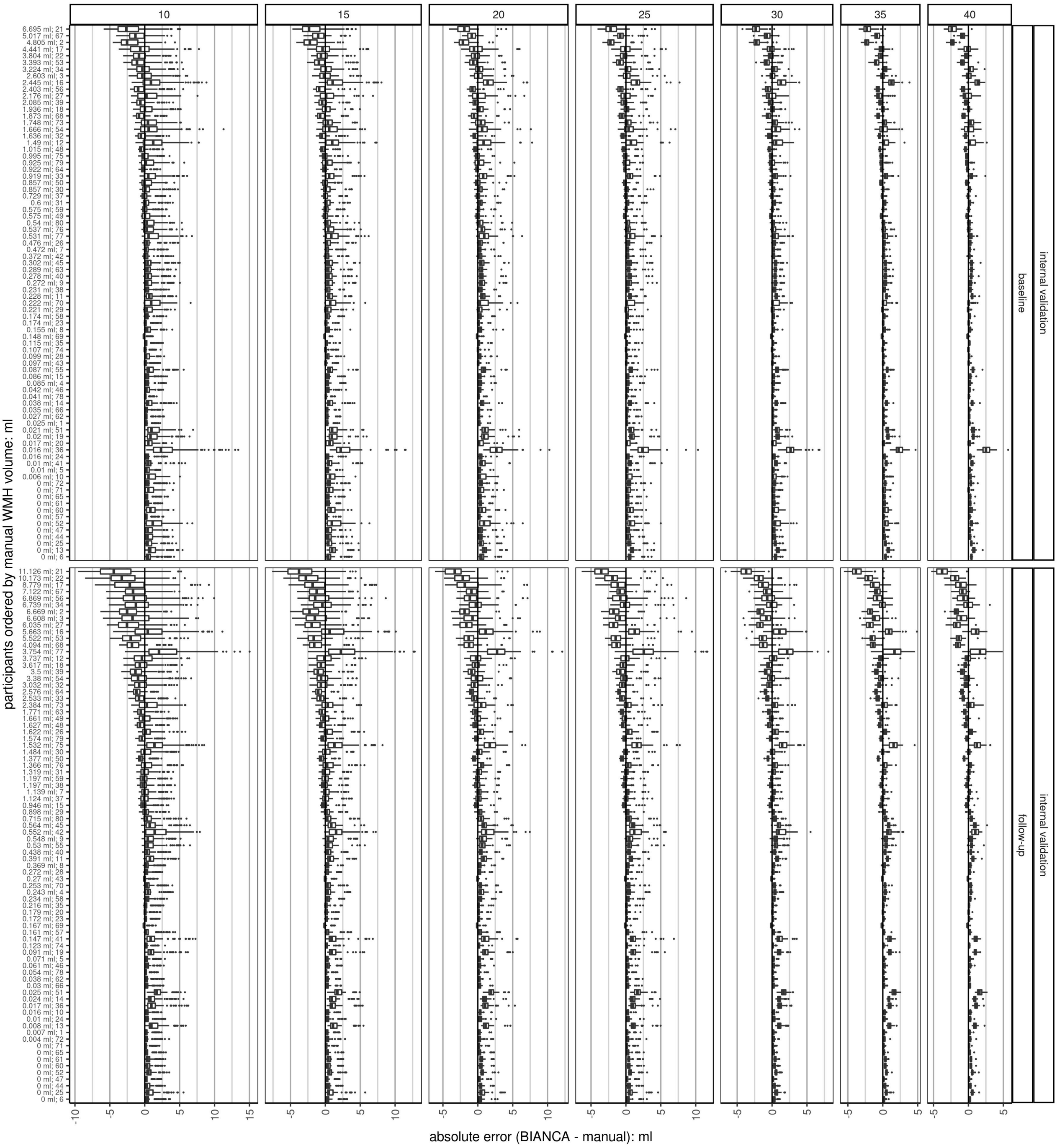

Figure S11. Intra-subject effects (internal validation set), sorted by manual lesion volume: Boxplots of each subject (n = 80, 2 time-points) per internal validation set (n = 2) and sample size (n = 7) at threshold 0.8. Shown are boxplots of the absolute error of all models for each participant. Each dot is a single observation. The black line indicates the ideal absolute error (BIANCA - manual volume) of 0 ml. Absolute errors greater than 0 ml show an overestimation of BIANCA, while absolute errors smaller than 0 ml show an underestimation.The figure shows two main systematic effects: (1) The higher the sample size, the higher the chance to train a model with a low deviation from the gold standard (smaller range of outliers and IQR), reflecting a more robust prediction. (2) Regarding accuracy: The higher the lesion volume, the more likely BIANCA underestimates the lesion volume. Moreover, mean absolute errors of some (random) observations deviate from the cohort throughout all sample sizes. This could hint towards effects of a certain anatomy, that lead to a general under- or overestimation by BIANCA. Please note: The scale of the x-axis is the same across sample sizes, but the x-axis is truncated to cover only the area of existing values.

0.2.7 Dice similarity index

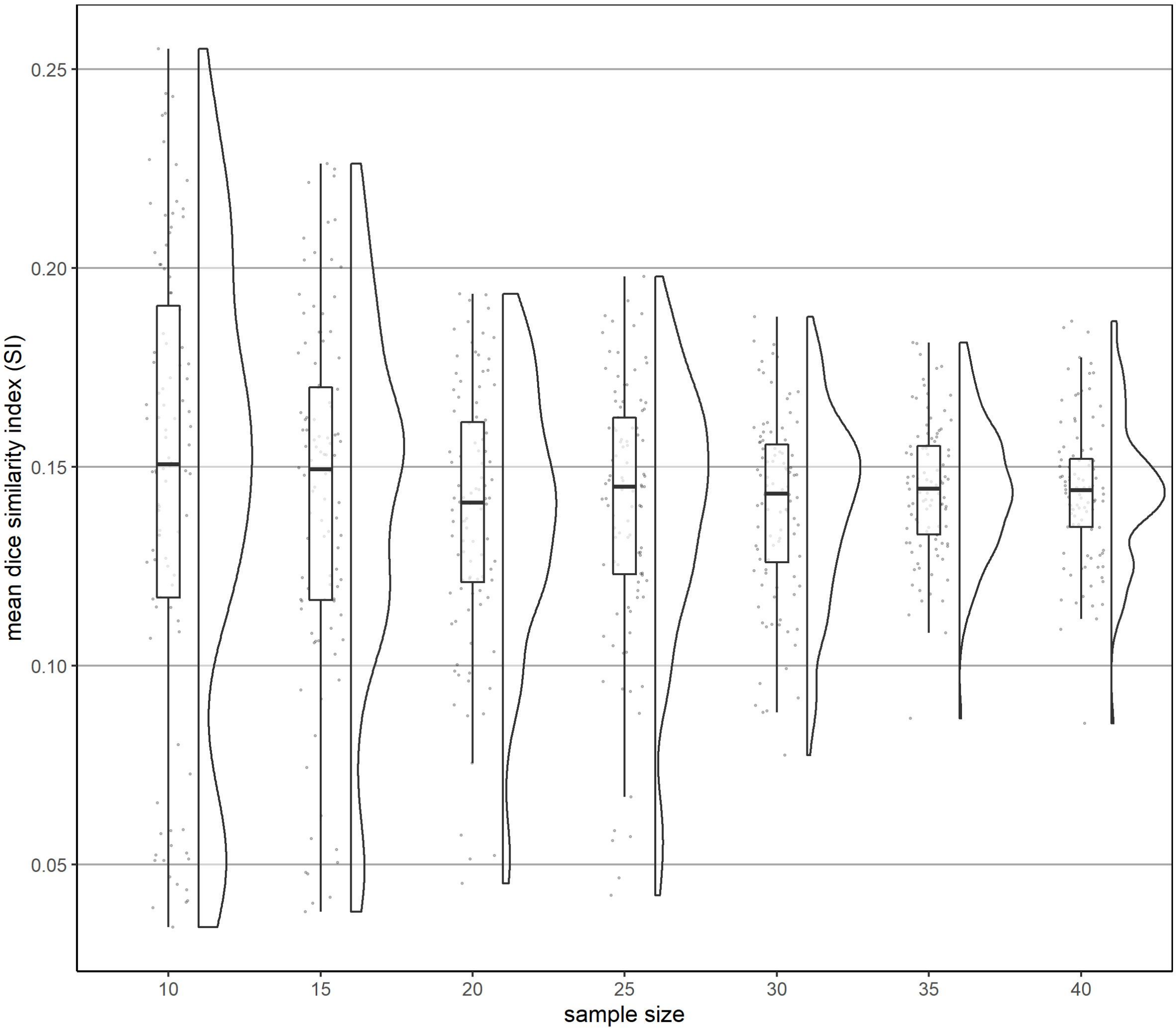

Figure S12. Raincloud plots of Dice similarity index (external validation set) stratified by sample size (n = 7) at threshold 0.8. Shown are raincloud plots (Allen et al., 2019) of the mean Dice similarity index (SI) for each model. Each dot is the mean SI of a model single model. The plots are stratified horizontally by sample size (n = 7). The higher the sample size, the more robust the SI, indicated by a decreasing IQR and range. The accuracy is generally low with a mean of 0.14 (SD 0,18) for a training sample size of n = 40.

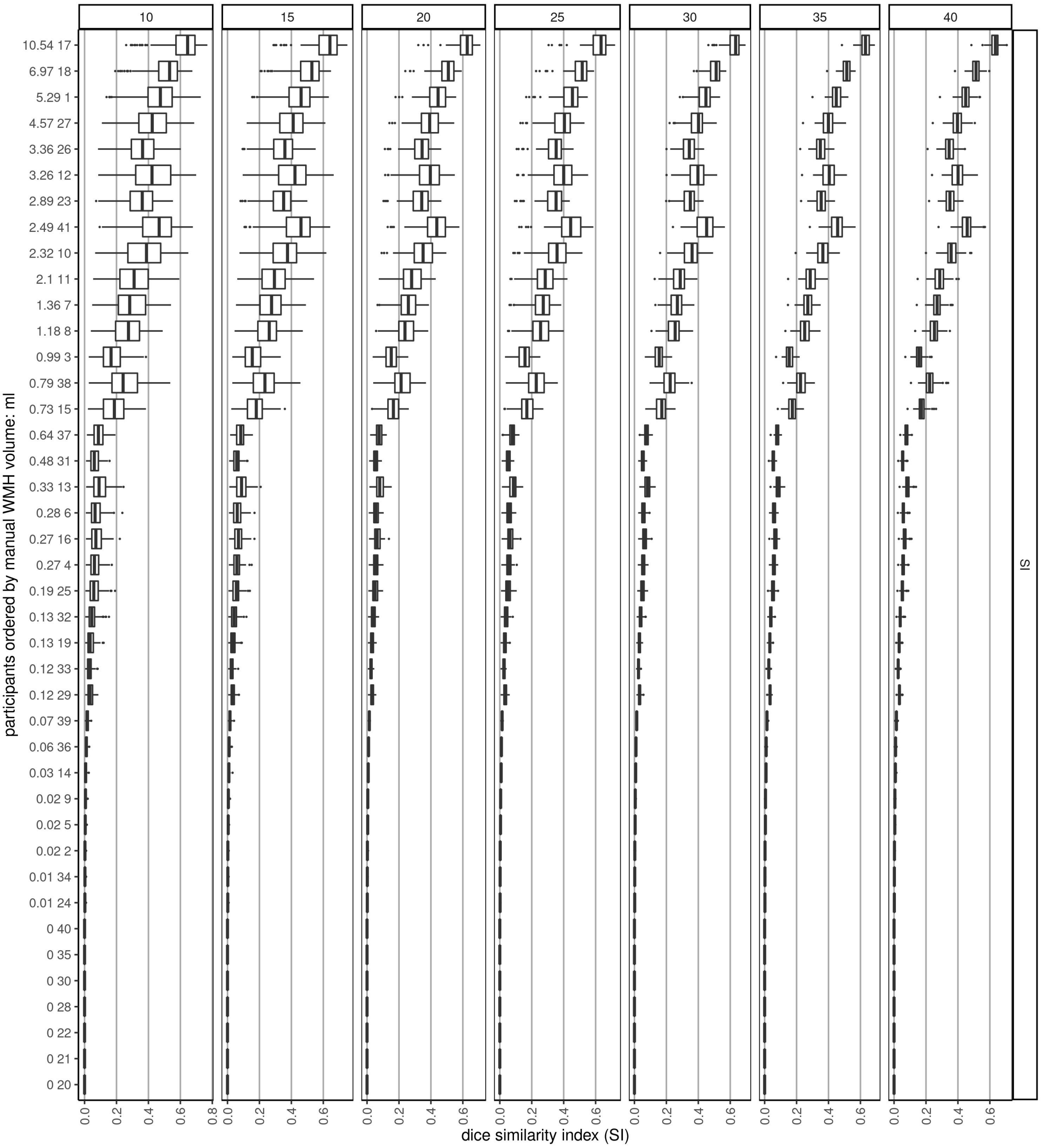

Figure S13. Dice similarity index (external validation set): Boxplots of the Dice similarity index (SI) stratified by subject (n = 41) and sample size (n = 7) at threshold 0.8. Shown are boxplots of the Dice similarity index (SI) for each participant and all models. Each dot is a single observation. The plots are stratified horizontally by sample size (n = 7) and ordered vertically by lesion volume. The scale of the x-axis is always the same, but truncated if necessary to cover only the area of existing values. The higher the sample size, the more robust the SI, indicated by a decreasing IQR and range. Regarding accuracy: The Dice similarity index is higher for subjects with higher lesion volume, than for subjects with lower lesion volume.

REFERENCES

Allen, M., Poggiali, D., Whitaker, K., Marshall, T., and Kievit, R. (2019). Raincloud plots: a multi-platform tool for robust data visualization [version 1; peer review: 2 approved]. *Wellcome Open Research* 4. doi:10.12688/wellcomeopenres.15191.1
